# Alzheimer’s Disease Diagnosis and Biomarker Analysis Using Resting-State Functional MRI Functional Brain Network With Multi-Measures Features and Hippocampal Subfield and Amygdala Volume of Structural MRI

**DOI:** 10.3389/fnagi.2022.818871

**Published:** 2022-05-30

**Authors:** Uttam Khatri, Goo-Rak Kwon

**Affiliations:** Department of Information and Communication Engineering, Chosun University, Gwangju, South Korea

**Keywords:** Alzheimer’s disease, rs-fMRI, brain network, DMN, SN, features selection, machine learning, SVM

## Abstract

Accurate diagnosis of the initial phase of Alzheimer’s disease (AD) is essential and crucial. The objective of this research was to employ efficient biomarkers for the diagnostic analysis and classification of AD based on combining structural MRI (sMRI) and resting-state functional MRI (rs-fMRI). So far, several anatomical MRI imaging markers for AD diagnosis have been identified. The use of cortical and subcortical volumes, the hippocampus, and amygdala volume, as well as genetic patterns, has proven to be beneficial in distinguishing patients with AD from the healthy population. The fMRI time series data have the potential for specific numerical information as well as dynamic temporal information. Voxel and graphical analyses have gained popularity for analyzing neurodegenerative diseases, such as Alzheimer’s and its prodromal phase, mild cognitive impairment (MCI). So far, these approaches have been utilized separately for the diagnosis of AD. In recent studies, the classification of cases of MCI into those that are not converted for a certain period as stable MCI (MCIs) and those that converted to AD as MCIc has been less commonly reported with inconsistent results. In this study, we verified and validated the potency of a proposed diagnostic framework to identify AD and differentiate MCIs from MCIc by utilizing the efficient biomarkers obtained from sMRI, along with functional brain networks of the frequency range .01–.027 at the resting state and the voxel-based features. The latter mainly included default mode networks (amplitude of low-frequency fluctuation [ALFF], fractional ALFF [ALFF], and regional homogeneity [ReHo]), degree centrality (DC), and salience networks (SN). Pearson’s correlation coefficient for measuring fMRI functional networks has proven to be an efficient means for disease diagnosis. We applied the graph theory to calculate nodal features (nodal degree [ND], nodal path length [NL], and between centrality [BC]) as a graphical feature and analyzed the connectivity link between different brain regions. We extracted three-dimensional (3D) patterns to calculate regional coherence and then implement a univariate statistical *t*-test to access a 3D mask that preserves voxels showing significant changes. Similarly, from sMRI, we calculated the hippocampal subfield and amygdala nuclei volume using Freesurfer (version 6). Finally, we implemented and compared the different feature selection algorithms to integrate the structural features, brain networks, and voxel features to optimize the diagnostic identifications of AD using support vector machine (SVM) classifiers. We also compared the performance of SVM with Random Forest (RF) classifiers. The obtained results demonstrated the potency of our framework, wherein a combination of the hippocampal subfield, the amygdala volume, and brain networks with multiple measures of rs-fMRI could significantly enhance the accuracy of other approaches in diagnosing AD. The accuracy obtained by the proposed method was reported for binary classification. More importantly, the classification results of the less commonly reported MCIs vs. MCIc improved significantly. However, this research involved only the AD Neuroimaging Initiative (ADNI) cohort to focus on the diagnosis of AD advancement by integrating sMRI and fMRI. Hence, the study’s primary disadvantage is its small sample size. In this case, the dataset we utilized did not fully reflect the whole population. As a result, we cannot guarantee that our findings will be applicable to other populations.

## Introduction

With the increase and the prevalence of age-related mental decline, researchers have been increasingly interested in studying pathological and regular aging in an attempt to identify early markers of neuronal disease. Indeed, due to the high expense and pharmaceutical burden of progressive disorder on the national healthcare system, research targeted at providing a timely and differential assessment of these disorders is essential. Alzheimer’s disease (AD) is the most prevalent neurodegeneration disease in the world, impacting millions of individuals (2020 Alzheimer’s disease facts and figures, 2020). The identification of precise and accurate biomarkers of early AD advancement will aid researchers and doctors in the development of novel medications and the monitoring of their efficacy, as well as reduce the time and expense of clinical examination. The National Institute of Neurologic and Communication Disorders and Stroke and the Alzheimer’s disease and Related Disorders Association (NINCDS-ADRDA) created clinical diagnostic guidelines for AD based on the binary method in the 1980s. The importance of cognitive deterioration in the detection of AD is mentioned in this technique (McKhann et al., 1984). Later, neuropathological evidence in the form of neurofibrillary tangles and senile plaques (Hyman and Trojanowski, 1997) was introduced. AD diagnostic regulation was enhanced in 2011 by the National Institute on Aging-Association Alzheimer’s Group. Additional features can be obtained by measuring cerebrospinal fluid (CSF), neurogenetic approach, tau, amyloid, and neuronal damage features as assessed by neuroimaging analysis, including MRI, positron emission tomography (PET), and functional MRI (fMRI). The use of biomarkers, such as the Mini-Mental State Examination (MMSE) score, MRI biomarkers (such as normalized whole-brain volume and hippocampal volume), and CSF biomarkers (amyloid–42, tau), as well as combined metabolic disorders, to detect AD and predict mild cognitive impairment (MCI) conversion shows promising future (van Maurik et al., 2017). MRI and PET imaging alterations allow for the determination of atrophic areas and amyloid/metabolism indicators (Zhang et al., 2011; Garali et al., 2018), allowing for the detection of AD even at an early stage (Jack et al., 2019). Because of the non-invasive nature of MRI, a lot of work has gone into improving the MRI processing scheme in order to uncover MRI-associated markers that may be used to increase the efficiency of Alzheimer’s diagnosis. There is a lot of evidence that various anatomical brain areas are damaged at different stages of the pathology, with the amygdala, hippocampus, and entorhinal cortex being the first to be impacted. Despite the fact that these areas are responsible for AD, they have yet to be thoroughly investigated. The hippocampal sub-regions are widely recognized to have a key part in the short-to-long-term memory assimilation process. The hippocampus region is more likely to be the first section of the brain to deteriorate. Furthermore, clinical research has revealed that the hippocampus region is one of the most often utilized and effective biomarkers for detecting the transition from MCI to AD (Platero et al., 2019; Liu et al., 2020). Despite this, due to the low resolution of MRI, it is commonly treated as a single entity. With major advancements in high-resolution MRI image data acquisition techniques, new possibilities for studying specific hippocampal sub-regions have emerged. CA1 volume measures were found to be more sensitive than total hippocampal volumetry for detecting structural alterations in the early stages of AD (Zheng et al., 2018). Hippocampus sub-regions have also been connected to age-related memory loss and certain features of memory patterns (Zheng et al., 2018). As a result, early identification of AD or its prodromal stage, MCI (Petersen, 2004) is critical for consistent and effective diagnosis, that can assist to slow the course of the disease. As an intermediate stage of Alzheimer’s, individuals with MCI are commonly marked by a cognitive and functional decline in the regular aging process and is affected by memory decline without the disorder (Petersen, 2004; Angelucci et al., 2010), which is commonly characterized by the overall decline in cognition on various brain regions. MCI is considered as prior AD (Petersen, 2004). Individuals with MCI develop Alzheimer’s subsequently, wherein symptoms emerge over the period of 2–3 years on average (Lopez et al., 2012). An eventual community support analysis in the old individual indicated that the transition rate of MCI from Alzheimer’s to various forms of dementia is approximately 10–15% every year (Wei et al., 2016). Previous research advises some individuals cannot transit to Alzheimer’s, and quite continue in stable form clinically for a long time, which is considered as stable MCI (MCIs), whereas MCI that converts to AD is referred to as MCIc. Previous research from Alzheimer’s analysis advocates the hypothesis that Alzheimer’s is commonly characterized by a functional disconnection of the neuronal pattern and functional connectivity (FC) of various brain areas, which is also shown in the initial stage of MCI or even before the transition to Alzheimer’s (Bishop et al., 2010; Clem et al., 2017; de Vos et al., 2018).

Biomarkers obtained from various imaging techniques, such as PET, resting-state fMRI (rs-fMRI), and structural MRI (sMRI), have shown beneficial aspects in the MCI and AD diagnosis (Ju et al., 2019). Particularly, the fMRI technique presents a wide analysis platform to quantify the functional patterns of the brain by calculating the correlation among intrinsic blood-oxygen-level-dependent (BOLD) frequency variation in multiple brain areas at resting state. Being sensitive to various brain region spontaneous neuronal activity, BOLD signal can be therefore utilized as an effective noninvasive biomarker for analyzing neuronal disease at a whole-brain level such as Alzheimer’s. FC, which gives the temporal interaction of BOLD frequency among various brain areas, can reveal how structurally isolated and functionally related brain regions communicate. Therefore, a functional network study utilizing fMRI images will serve an immense potentiality for automated disease diagnosis. A large literature has analyzed AD-generated alteration in functional networks on rs-fMRI (Hojjati et al., 2017; Zhang et al., 2019). The rs-fMRI presents the insight on a dynamic imaging modality for pathological identification of FC not only in an individual with Alzheimer’s but also in those with other neuropsychiatric or neurological disorders (Greicius, 2008). Evidence from previous studies indicates that functional relation at the resting state shows the connection link of work-related knowledge (Ito et al., 2017), in which functional networks have proven to be highly valuable and sensitive markers for Alzheimer’s (Sheline et al., 2010). Grieder et al. (2018) advise that loss in cognitive ability in AD individual are directly connected to the brain network complexity pattern. In a previous fMRI study, FC has been reported to indicate Alzheimer’s related cognitive impairment in older individuals with healthy cognition, AD, and MCI (Lin et al., 2018). Some fMRI analyses also indicated that the pathophysiology of Alzheimer’s is correlated with statistical alteration of regional spontaneous low-frequency BOLD variation coherence estimation in the relaxed phase. For voxel-wise analysis, the metrics used in these studies included regional homogeneity (ReHo) (Zang et al., 2004; He et al., 2007); the amplitude of low-frequency fluctuation (ALFF) (Zou et al., 2008; Li et al., 2017), and fractional ALFF (fALFF). These studies showed that the precuneus (PCu) and the posterior cingulate cortex (PCC) had the larger ReHo abnormality among Alzheimer’s as compared to cognitively normal individuals (*p* < 0.05). The fALFF along with ALFF analysis on fMR images (Han et al., 2011) suggested, individuals with MCI are characterized by reduced fALFF measure in the larger brain areas, which includes temporal cortices and occipital. The FC of rs-fMRI (Li et al., 2017), indicated, a brain area with major FC was highly presented in the default mode network (DMN) regions (Hafkemeijer et al., 2012; Zhang et al., 2020) and primarily affect the PCC and bilateral PCu (Dai et al., 2015). Both AD and aMCI have been found to target large-scale networks, including reduced DMN connectivity and increased salience network (SN) (Greicius et al., 2004; Zhou et al., 2010; Zhou and Seeley, 2014) connectivity, as well as aberrant connectivity between networks (Brier et al., 2012) in AD and disturbed connectivity in aMCI, especially in relation to the DMN (Lee et al., 2016; Yang et al., 2017; Zhang et al., 2017), using resting-state functional connectivity methods that quantify the temporal synchrony between brain regions. These are all statistically important discoveries for cluster-level comparison. Yet, the classification potential of the above-indicated biomarker identified the individual with MCI/AD into one of the categories (MCI/AD vs. healthy controls [HC]), which is a highly complex work as compared to the study of different groups (Rathore et al., 2017). Recently, another analysis recommended that the biomarkers retrieved from functional networks assessment and machine learning techniques using rs-fMRI provide an effective framework for accurate and efficient identification. Chen et al. (2011) utilized large-scale networks (LSN) technique with 95% area under the curve (AUC) to classify patients into amnestic MCI (aMCI) and cognitively healthy. Challis et al. (2015) presented the GP-LR technique with SVM and obtained an accuracy of 75% to diagnose healthy individuals from aMCI. Khazaee et al. (2015) utilized time series from a brain network with linear SVM as a classifier to identify disease individuals, their experimental result achieved 100% accuracy on classification. This could be due to the limited number of sample and their feature reduction technique which was the single-variable Fischer score method. In another study, they utilized features obtained both spatial and temporal variation from dynamic connectivity networks (DCNs). Finally, they combined it as a feature to evaluate the multi-kernel technique along with manifold regularization multi-task feature learning and obtained 78.8% of accuracy for the identification of EMCI and LMCI (Jie et al., 2018). It has been proven that a functional graph measure with a machine learning technique using rs-fMRI can precisely identify individuals with MCI, individuals with Alzheimer’s, and healthy control (Xiang et al., 2013).

However, large of the literature collected MCIs and MCIc groups into a single MCI group (He et al., 2008; Khazaee et al., 2015), and very few works of literature have analyzed the potentiality of rs-fMRI to identify differences among the two groups (Khazaee et al., 2015). In addition, Zuo et al. (2010) categorized the BOLD frequency into five frequency bands. Brain function in individuals with AD and MCI was significantly different at hippocampus, medial prefrontal, and posterior cingulate regions in these frequency bands. From this framework, we aimed to assess the potency for diagnostic classification to differentiate MCIs and MCIc, along with other groups, by using the biomarkers obtained from sMRI and functional brain networks during the resting state. Based on the classification results, to discover highly sensitive biomarkers, we can recognize accurately and precisely why sensitive biomarkers in the brain altered with disease advancement. We hypothesized that providing cognitive training and appropriate treatment for an individual’s highly sensitive brain area at various phases in disease advancement can avert the growth of Alzheimer’s conversion. It is better to observe that large of the abovementioned rs-fMRI technique utilized only time-series networks to compare different groups. However, using only time series for a feature vector obtained by rs-fMRI modality is possibly not precise to present the spatiotemporal pattern of the whole brain (He et al., 2008).

Volume alterations in the hippocampus and amygdala are considered a primary feature of AD and are utilized as a diagnostic indication. In Alzheimer’s patients, hippocampal and amygdala atrophy generally spreads to other parts of the brain (Josephs et al., 2017; Feng et al., 2018). Anatomical MR imaging can be used to visualize the pattern of hippocampus amygdala, cortical, and subcortical atrophy. Which is important in the clinical diagnosis of AD (Feng et al., 2018). Therefore, the fundamental aim of this framework was to analyze the sMRI and rs-fMRI data to their full potential by combining hippocampal sub-volume, amygdala nuclei volume of sMRI, brain networks, and multi-measure voxel-based features of rs-fMRI to identify AD. Firstly, the cortical and subcortical segmentation was performed with Freesurfer (version 6), and then hippocampus subfield and amygdala nuclei volume segmentation was performed with Freesurfer’s segmentHA_T1.sh function (Fischl, 2012). Secondly, we processed the signal into the 0.01–0.027 Hz frequency band at the resting phase. Thereafter, we created a brain network by evaluating Pearson’s correlation coefficients among time series of the entire brain region. Afterward, we performed a threshold operation to obtain a binary undirected brain network. Subsequently, we obtained graph elements, such as global efficiency, local efficiency, characteristic path length, clustering coefficient, and SmallWorlds, to calculate the parameters of functional brain networks. Likewise, we obtained maps of three-dimensional (3D) regional coherence (fALFF, ALFF, ReHo, and DC) for each patient. After this we implemented univariate statistical two-sample *t*-tests for the entire 3D-brain area among training classes to obtain an analysis mask that preserved the original set of significant voxel-based features, generating notable differences in any one of the voxel measures, that is, fALFF, ReHo, FALFF, and DC. In this study, we also applied brain networks and voxel features separately, and finally, we combined both sMRI and rs-fMRI features. At the feature selection stage, we implemented and analyzed three different feature selection techniques to obtain the optimal features. To achieve unbiased classification performance, SVM with the cross-validation method (CV) was implemented as a classifier. More importantly, we also compare the performance of our model with the ensemble learning approach using Random Forest (RF) classifiers.

## Materials and Methods

### Participants

Data used in the preparation of this article were obtained from the AD Neuroimaging Initiative (ADNI)^1^ database. The ADNI was launched in 2003 as a public-private partnership, led by Principal Investigator Michael W. Weiner, MD. The primary goal of ADNI has been to test whether serial MRI, PET, other biological markers, and clinical and neuropsychological assessment can be combined to measure the progression of MCI and early AD. Individual inclusion criteria for subjects were mentioned in the ADNI conduct. The included individuals were between the ages of 53 and 93 years. All individuals were able and willing to endure all test procedures, along with neuroimaging, and admitted to a longitudinal investigation. Psychoactive treatment was not included in the assessment. In this framework, we obtained data for all individuals accessible on the ADNI webpage. In total, 213 individuals were included as either AD (*n* = 63), MCIs (*n* = 37), MCIc (*n* = 45), or HC (*n* = 68), matched with age and sex ratio. Group categories were sorted through the functional activities questionaries (FAQ) record between 0 and 4, the Mini-Mental State Examination (MMSE) record 26–30, and the Geriatric Depression Scale (GDS) record between 0 and 4. For the MCI case, the FAQ record was 0–16, the MMSE record was 24–30, and the GDS record was 0–13. For the MCIc case, the FAQ record was 0–18, the MMSE record was 18–30, and the GDS record was 0–10.

For the AD case, the individual had a global CDR score of 1, an FAQ score of 3–28, an MMSE score of 14–24, and a GDS score of 0–7. Individuals with MCI who had been followed for less than 18 months and did not convert were not included in this study. Table 1 presents the demographic report of individuals who participated in the study, including the sex ratios and mean age for each category. Statistically significant differences in the clinical and demographic features were assessed among these categories, Student’s *t*-test was utilized at a.05 significance level. We did not notice significant changes (*p* > 0.05) among the category in sex ratio or age. For unbiased evaluation of performance, group classifications were randomly shuffled and split into k subgroup. For model evaluation training, datasets were trained and classification performance was measured on diagnostic sensitivity, specificity, F1-score, and Cohen’s Kappa index on the independent testing set. Sex and age distribution were preserved on the splitting procedure.

**TABLE 1:** Neuropsychological and demographic characteristics of participants.

Group	AD (*n* = 63)	MCIs (*n* = 37)	MCIc (*n* = 45)	HC (*n* = 68)
Sex (M/F)	37/26	15/22	27/18	33/35
Age	74.51 ± 7.18	73.32 ± 7.58	73.81 ± 7.91	76.37 ± 7.13
FAQ score	19.95 ± 7.73	1.72 ± 2.15	7.23 ± 7.18	0.14 ± 0.37
NPI-Q score	4.85 ± 5.13	1.87 ± 1.75	2.78 ± 2.85	0.37 ± 0.83
GDS score	2.32 ± 2.87	1.28 ± 1.05	2.35 ± 2.95	1.19 ± 1.95
MMSE score	19.95 ± 5.15	29.12 ± 1.03	25.17 ± 3.45	29.25 ± 1.75
CDR	0.94 ± 0.27	0.50 ± 0.0	0.50 ± 0.28	0.00 ± 0.13

*Values are means or numbers ± standard deviations.*

*MMSE, mini-mental examination; NPI-Q, neuropsychiatric inventory questionnaire; FAQ, functional activities questionaries; GDS, geriatric depression scale.*

### Structural MRI Preprocessing

From the ADNI webpage, we obtained 1.5-T T1-weighted MR images. The MRI scans were collected using Philips, GE, or Siemens Medical system scanners from data centers. Because the acquisition methodology of each scanner was different, ADNI performed an image normalization step. Image corrections encompassed calibration, image geometry distortion due to gradient non-linearity (grad-warp), a decrease in intensity non-uniformity due to waves, or a decrease in residual intensity non-uniformity of the 1.5-T scans utilized by ADNI. On the ADNI website, we can get more information regarding the sMRI. All scans had a resolution of 176×256×256 and were spaced 1 mm apart. We used the Freesurfer^2^ (version 6) (Fischl, 2012) toolbox to pre-process the collected sMRI images in our experiment.

### Resting-State Functional MRI Image Acquisition

Philips Medical sMRI scanner of 3 T was utilized to obtain the fMR images. The rs-fMR images were acquired through the ADNI webpage. At the time of image acquisition, patients were asked to not to think, to lie in the scanner, and to relax. The parameters sequences were as follows: TR = 3,000 ms, plus sequence = GR, flip angle = 800°, TE = 30 ms, data matrix = 64 × 64, slice thickness = 3.33 mm, pixel spacing *X* = 3.31 mm and *Y* = 3.31 mm, axial slices = 48, time points = 140, and no slice gap.

### Resting-State Functional MRI Preprocessing

Image processing procedures were achieved by utilizing Data Processing Assistant for Resting-State fMRI (DPARSF) (Yan and Zang, 2010) containing Statistical Parametric Mapping (SPM)^3^ and Resting-State fMRI Data Analysis Toolkit (REST)^4^. For stabilization and adaptation of individuals, participants’ first 10 time points were removed followed by correction of the last slice time. To compensate for the effect of head motion realignment, spatial transformation of a six-parameter rigid body was used. All spatial motion displacements were performed for <3 mm and <30° of rotation in each direction. Further, co-registration of rs-fMR images to 3D-T1 structural high-resolution images was carried out. The Montreal Neurological Institute (MNI) space was undertaken to normalize 3D-T1 structural MR images by non-linear wrapping based on Diffeomorphic Anatomical Registration *via* Exponential Lie Algebra (DARTEL). Individual fMRI images were then spatially normalized to the MNI field utilizing the parameters collected from the normalization of the structural image and simultaneously resampling them into 3-mm isotropic voxels. The 6-mm full-width half-maximum Gaussian kernel was utilized on normalized individual rs-fMR data. Linear detrending and band-pass filtering at 0.01–0.027 Hz were performed. The 6-mm FWHM Gaussian kernel for spatial smoothing was implemented. The six head motion parameter, the global mean signal, the white matter (WM), and the CSF signal were discarded as nuisance variance to decrease the motion effects and non-neuronal BOLD variation (Hojjati et al., 2018). Similarly, for voxel-based features, mask images were obtained according to the subject specialized normalized T1 anatomical images. The voxel measures within the mask were utilized for the analysis. The mask images obtained were utilized for developing various testing in further investigation and analyses.

### Proposed Framework

**FIGURE 1:**
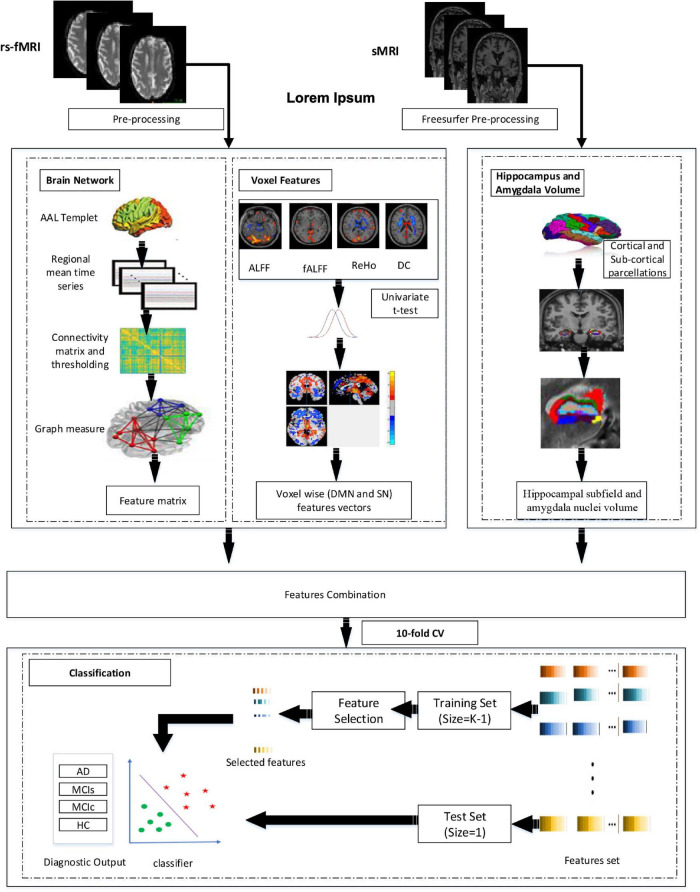
Overview of the proposed framework.

Figure 1 represents the proposed procedure used in this framework. The first step of the framework was to prepare and process the sMRI (hippocampal subfield, amygdala volume) and rs-fMRI images to obtain the corresponding time series and whole-brain 3D measurements. From time-series data, we constructed the brain network. Similarly, we obtained the fALFF, ReHo, ALFF, DC, and SN feature vectors from 3D measures. From brain network construction, we obtained a nodal degree (ND), betweenness centrality (BC), and the nodal path length (NL). Similarly, for the voxel-based 3D structural model, we retrieved a 3D mask that determined a set of “effective” voxels to conduct statistical univariate *t*-tests. Thereafter, we combined the hippocampal-amygdala subfield volume (sMRI), the brain network, and voxel-based feature vectors (rs-fMRI) for the final classification. We then applied the feature reduction technique on the integrated training features set to choose highly significant features vectors to train the SVM and the RF classifiers. We finally obtained significant feature rank and fed the feature vectors as the training sample, and we also obtained the testing sample for classification. Owing to the limited size of the dataset, we utilized a CV of 10-fold to validate the diagnosis performance of the classification for the proposed framework. While performing the 10-fold CV, 90% of the total sample was utilized for the training process and the remaining 10% for testing.

### Hippocampal Subfield and Amygdala Nuclei Volume

**FIGURE 2:**
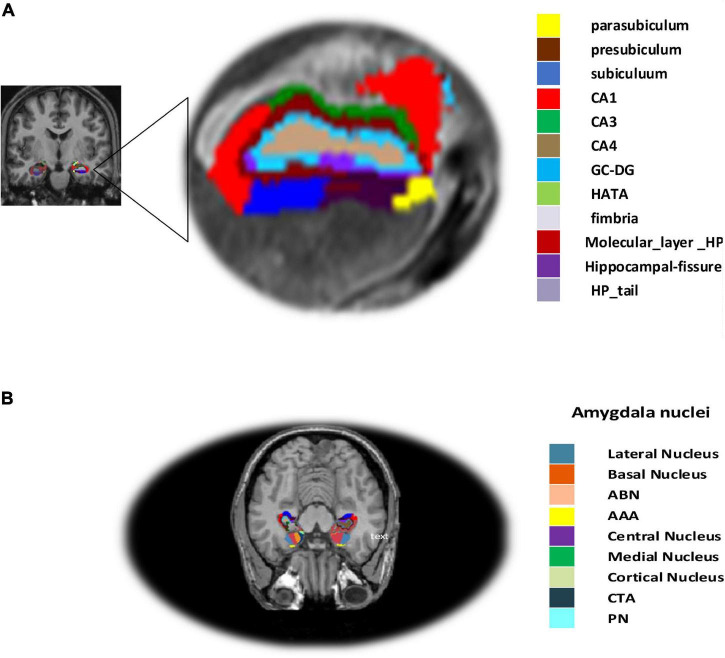
**(A)** Structural MRI (sMRI) of hippocampal subfield and **(B)** amygdala nuclei volume.

Hippocampal subregions in MRI have been demonstrated to play a role in predicting AD in those with moderate symptoms (van Maurik et al., 2017). It is more crucial to assess the sub-volume of the hippocampus in order to compute atrophy measures on the hippocampal subfield more precisely and to identify AD in individuals with MCI as well as normal controls (Zheng et al., 2018). Hippocampal segmentation was carried out utilizing the Freesurfer (Fischl, 2012) software in our procedure. First, the cortical and subcortical segmentation was performed with Freesurfer (version 6), and then the hippocampal subfield and amygdala nuclei volume segmentation were performed with Freesurfer’s segmentHA_T1.sh function (Fischl, 2012), and details can be found at^5^. Hippocampus atrophy is thought to be a key indicator of AD (Sørensen et al., 2017). The hippocampal subregion parcellation approach, provided by Freesurfer, was finally used to estimate hippocampal subregions and amygdala nuclei subfields (version 6). This program creates a computational parcellation of the amygdala and hippocampal regions using an atlas-based probabilistic Bayesian interface and ultra-high resolution *ex-vivo* MRI imaging data (0.1–0.15 mm isotropic). Simultaneous segmentation of both structures ensures that there is no overlap between them and that there is no chance of a gap between them (Saygin et al., 2017). The subiculum, the presubiculum, the parasubiculum, the cornu amonis fields 1, 2/3, and 4 (referred to as CA1, CA3, and CA4), the granule cell sheet of the Dentate Gyrus (DG), a transition of Hippocampus-Amygdaloid Area (HATA), the fimbria (a white matter area), the molecular coat of DG, the fissure region of the hippocampus, and the tail of the hippocampus are the 12 regions, as illustrated in Figure 2. Similarly, the accessory basal and basal, the central medial, the lateral, the cortical, and the anterior amygdala regions, the para laminar nucleus, and the Cortico-Amygdaloid Transition Area (CTA) are the nine subregions of the amygdala.

### Functional Network Construction

The brain network nodes were constructed by parcellation of the entire brain into 90 regions of interest by utilizing the automated anatomical labeling (AAL) template that gives an entire functional division of the cortex (Tzourio-Mazoyer et al., 2002). The time series obtained inside every voxel of the 90 regions of interest were averaged, which produced signals that served as the nodal features. Pearson’s correlation coefficient among the time series of the entire brain region was used to construct the value of the edges for networks. Then, Fischer’s *r*-to-*z* conversion was practiced on the rough random connectivity matrix to enhance the partial correlation coefficient uniformity (Risacher et al., 2009; Wee et al., 2012). The obtained matrix is symmetrical diagonally with zero value (Zhan et al., 2013). The sparsity threshold was utilized to define the value of the possible edges on an individual’s brain networks. The threshold serves as the connection cost for networks, which is explained as the ratio of the total possible number of connections to the suprathreshold relation within the brain networks (Sanz-Arigita et al., 2010). There is no straightforward approach to define a single sparseness threshold because various sparseness drive various analysis outcomes (He et al., 2009; Hojjati et al., 2018). This experiment examined each network at costs ranging from 5 to 25% at 1% intervals. In addition, we also carried out an analysis over various threshold values to examine the excellent threshold value (Fornito et al., 2010). To create efficient network parameters statistically fascinating variations in network parameters among different classification groups on various sparsity levels were measured.

### Brain Network Feature Extraction

Entire brain networks parameters were calculated and evaluated utilizing the Matlab 2019a^6^ program and matlab_bgl^7^. All graph matrices were evaluated using the undirected connectivity matrix on 0.01–0.027 Hz frequency band. To retrieve dynamic features and eliminate largely redundant matrix features, we computed five global graph parameters for the undirected graphs. These five global graph parameters are as follows: local efficiency, global efficiency, clustering coefficient, characteristic path length, and small-worldness (Tan et al., 2019). In the feature extraction section, we extracted brain network features for 270 nodal features of the brain network, in which ND, NL, and BC were calculated for further study and for the classification framework. In short, for the obtained node i, ND, NL, and BC were defined as follows:


(1) $$L_{i}=\frac{\sum_{j\neq i\in V}L_{i\,j}}{\left(V-1\right)}$$

(2) $$K_{i}=\sum_{j\in V}b_{i\,j}$$

(3) $$B_{i}=\sum_{i\neq j\neq m\in V}\frac{S_{j\,m}\,\left(i\right)}{S_{j\,m}}$$

where *L*_*ij*_ represents the minimal number of edges among nodes *i* and *j*, V represents the range of the graph, *b*_*ij*_ represents the network structure among nodes *i* and *j*, *S*_*jm*_ represents producing the shortest path length number among nodes *m* and *j*, *S*_*jm*_(*i*) where indicated the shortest path number over the node *i* among nodes *m* and *j*. Possibly, path length *L*_*i*_ calculates the pace of the message that is carried *via* a specific node, each node degree *K*_*i*_ corresponds to the connected link number to the particular node; the larger the value of *b*_*i*_, the higher the meaning of node *i* to the communication link within the networks, which reflect the information interaction level in the brain network.

### Voxel-Wise Features Extraction

In this study, we illustrated voxel-base feature estimation from rs-fMR utilizing the REST toolbox pipeline. These voxel features can be classified into DMN (ReHo, fALFF, and ALFF), DC, and SN. We utilized the ReHo voxel to examine the regional brain activity amid the phase of the brain at resting. The Kendall’s Coefficient of Concordance (KCC) approach was utilized to calculate the voxel-wise features. Individual ReHo maps were obtained from all brain voxels for individual subjects. A higher ReHo value was obtained for the voxel consisting of its closest neighbors, and brain voxel features were obtained for a larger regional coherence within a cluster. Larger analyses in recent literature indicated the potentiality of ReHo in recent clinical practice (Zang et al., 2004; He et al., 2007). Similarly, the ALFF measure estimates the regional spontaneous activities of the brain, and fALFF is an improved version of ALFF. The time series were transformed and filtered to the frequency region utilizing a fast Fourier transform (FFT) followed by its corresponding power range. The fALFF is a modified domain of ALFF, which is characterized by the average amplitude ratio within the low-frequency range. A large body of literature on the brain has indicated the unusualness level of the particular signal in brain regions for a disease class as related to a control class while employing statistical univariate tests (Arbabshirani et al., 2017). Recently, many works of literature have utilized *t*-tests to calculate descriptive biomarkers from neuroimaging for machine learning (Chaves et al., 2009; Wee et al., 2012). The main results of the statistical analysis tests are generally carried out using *p*-values. We generated a diagnostic feature estimation, that is, ReHo, fALFF, ALFF, and DC, among two classification groups at the threshold value of *p* < 0.05. The correlation cluster size on the defined threshold (*p* = 0.05) associated with the respective voxel *p*-value was determined by Monte–Carlo simulations using the AlphaSim tool embedded in REST to calculate the cluster value and the cluster size. Similarly, for SN, we utilized a well-validated region of interest (ROI) that included 12 posterior and 7 anterior SN nodes accessible at^8^ and were extracted using independent component analysis (Shirer et al., 2012).

### Features Selection Techniques

The number of features per subject, as in the neuroimaging study, is extremely high in comparison to the number of patients, a phenomenon known as the curse of dimensionality. Furthermore, dealing with a large number of features might be problematic because of the computational limitations of dealing with high-dimensional data, which can lead to overfitting. Feature selection is a step that comes before the classification problem and helps to minimize the dimensionality of a feature by choosing the right features and leaving out the wrong ones. This stage reduces the computing time for the training and testing datasets, speeding up the classification process and improving classification accuracy. To remove duplication and dependence, we first normalized the extracted features using the standard scalar function from the Scikit-learn module (Pedregosa et al., 2011), which modifies the dataset in such a manner that its distribution has a mean of 0 and a unit variance of 1. Features selection techniques utilized in this model are described below.

### Least Absolute Shrinkage and Selection Operation

The least absolute shrinkage and selection (LASSO) method (Tibshirani, 1996) is a dynamic process and is utilized to select the significant features set. This method is basically based on regularization and feature elimination. The LASSO technique helps to reduce the residual sum of squares present in the analysis by ordinary least square regression (OLS), which places a constraint on the absolute sum values of the design framework. LASSO measures model coefficients β by minimization of the function below:


(4) $$R\,S\,S_{L\,A\,S\,S\,O}\,\left(\beta_{i},\beta_{0}\right)=\underset{\beta }{a\,r\,g}\min\left[\sum_{i=1}^{n}\left(y_{i}-{\left(\beta_{i}\,x_{i}+\beta_{0}\right)}^{2}+\alpha \,\sum_{j=1}^{k}\left|\beta_{j}\right|\right)\right]$$

where *x*_*j*_ represent the data input at observation *j* and a vector *k*, and *n* represent the observation sample. *y*_*j*_ represent the observation response at *i*. α is a user-defined non-negative parameter for regularization that controls the penalty strength. If α is largely sufficient, then parameters are compelled to be zero, ultimately leading to generating only efficient features vector. When α tends to zero, the model turn to OLS with the most efficient features vector (Hanyu et al., 2010).

### Support Vector Machine-Recursive Feature Elimination

The support vector machine-recursive feature elimination (SVM-RFE) method is basically a multivariate wrapper technique based on the backward feature elimination technique, which precisely adopts a model and eliminates the less relevant feature vector till the specific number of relevant features is obtained. The ranking principle for the SVM-RFE is identical to the SVM technique. After this, the features that acquired the lowest rank is eliminated because it has the lowest response on evaluation, while the other feature vectors are selected for the SVM model in another iteration. All the irrelevant feature vectors were removed based on the repeated sequential procedure. Entire feature vectors are graded according to the elimination rank. A detailed explanation of the SVM-RFE technique can be explored in a preceding article (Guyon et al., 2002). In this study, after the implementation of SVM-RFE, the highly informative training feature vectors were kept which boosted cross-validated performance accuracy to train the classifiers.

### Joint Mutual Information

Mutual information (MI) can be applied to evaluate any arbitrary relation among random variables in the information theory (Kraskov et al., 2004). Truly, the MI among two arbitrary variables, X and Y, is a calculation of the measure of knowledge on Y given by X or, reversely, on X supplied by Y. If X and Y are independent, that is, if X has no message about Y and vice versa, then mutual information between them is zero. For two random variables, X and Y, MI is calculated as


(5) I⁢(X;Y)=H⁢(X)-H⁢(X/Y)=H⁢(Y)-H⁢(Y/X)=H⁢(X)+H⁢(Y)-H⁢(X;Y)

where H(.) is entropy, *H*(*X*/*Y*) and *H*(*Y*/*X*) represent conditional entropies, respectively, and similarly, *H*(*X*;*Y*) represent joint entropy for X and Y, which are calculated as


(6) $$H\,\left(X\right)=\int_{x}P_{X}\,\left(x\right)\,\log P_{X}\,\left(X\right)\,dx$$

(7) $$H\,\left(Y\right)=-\int_{y}P_{Y}\,\left(y\right)\,\log P_{Y}\,\left(y\right)\,dy$$

(8) $$h\,\left(X;Y\right)=-\int_{x}\int_{y}P_{X,Y}\,\left(x,y\right)\,\log P_{X,Y}\,\left(x,y\right)\,dx\,dy$$

where *P*_*x*_(*x*) and *P*_*y*_(*y*) represent marginal density value and *P*_*x*,*y*_(*X*,*y*) defined the joint probability density value for X and Y, correspondingly. The function which defines the marginal density are:


(9) $$P_{X}\,\left(x\right)=\int_{y}P_{X,Y}\,\left(x,y\right)\,dy$$

(10) $$P_{Y}\,\left(y\right)=\int_{x}P_{X,Y}\,\left(x,y\right)\,dx$$

By substituting Eqs. 6, 7, 8 in Eq.5, the MI equation will be


(11) $$I\,\left(X;Y\right)=\oint_{x}\int_{y}P_{X,Y}\,\left(x,y\right)\,\log\frac{P_{X,Y}\,\left(x,y\right)}{P_{X}\,\left(x\right)\,P_{Y}\,\left(y\right)}\,d\,x\,d\,y$$

The discrete form of the equation can be represented by the integration over summation in the date for all possible values. Therefore, estimation of *P*_*x*,*y*_(*x*,*y*) is required to calculate the Joint Mutual Information (JMI) between X and Y. The discrete form of JMI is represented by the below equation.


(12) $$I\,\left(X;Y\right)=\sum_{x\in X}\sum_{r\in Y}P_{X,Y}\,\left(x,y\right)\,\log\frac{P_{X,Y}\,\left(x,y\right)}{P_{X}\,\left(x\right)\,P_{Y}\,\left(y\right)}$$

when *F*_*k*_ is one of the traits in a set of traits {*F*_1_,*F*_2_,…,*F*_*k*_} and Y is an outcome that can be assumed by the trait, and the MI technique can pick the efficient trait. The process usually treats the trait as an independent random variable and is sort in a descending order based on their mutual information according to the obtained value Y, which selects the top n number of features. The process is conditioned by parsimonious and proper feature vectors that should (i) not be highly correlated among them and (ii) be individually relevant. JMI is shared among {*F*_1_,*F*_2_,…,*F*_*k*_} and Y is highlighted at Eq. 13, where *F*_*k*_ and Y represent the elements of *F*_*k*_ and Y, correspondingly.


$$I\,\left(F_{1},F_{2},\ldots ,F_{k}\right)=\sum_{f_{1}\in F_{1}}\sum_{f_{2}\in F_{2}}\ldots \,\sum_{f_{k}\in F_{k}}\sum_{y\in Y}$$

(13) $$P\,\left(f_{1},f_{2},\ldots ,f_{k},y\right)\,\log\frac{P\,\left(f_{1},f_{2},\ldots ,f_{k},y\right)}{P\,\left(f_{1},f_{2},\ldots ,f_{k}\right)\,P\,\left(y\right)}$$

A JMI feature elimination technique initializes from an empty feature of trait, which iteratively calculates *F*_*i*_*s* and is added to the empty set, creating the optimum increment measure in the JMI among the feature vectors and the results. JMI is considered to be a highly stable and a flexible feature reduction technique amid all the information-theoretic feature elimination techniques established until now.

### Random Forest Classifier

Random Forest is an ensemble learning approach, originally developed by Breiman (2001) to address regression and classification problems. The implementation of RF is based on the parameters set up, among which is the number of features in each branch and the number of trees. Previous studies noted that the optimum results could be obtained by setting default parameters (Immitzer et al., 2012; Zhang and Roy, 2017). However, studies conducted by Liaw and Wiener (2002) suggested that the larger the number of trees the more stable will the results be. In another study, Breiman (2001) noted that using more number of trees may not be beneficial for performance, but there is also no negative effect for the model. In another piece of literature, Feng et al. (2015) analyzed that, with the number of trees = 200, RF could reach precise results. Regarding the split parameter, many previous studies utilized the default parameter value $\sqrt{p}$; where *p* represented the number of predictor variables (Duro et al., 2012). However, in our model, we set a number of trees = 100, 200, 500, and 1000; split = 1:10 with a step size 1 to obtain the optimal performance. RF classifiers were implemented using the Scikit-learn Python library (Pedregosa et al., 2011).

### Support Vector Machine Classifier

As a supervised learning technique, SVM (Cortes and Vapnik, 1995) divides the classification group by finding the best hyperplane. By training data, SVM is trained in a given features space. Thereafter, that test dataset is classified according to its arrangement in the n-dimensional vector field. SVM has been practiced in numerous neuroimaging fields (Zhang et al., 2011; Collij et al., 2016) and is recognized as one of the highly robust machine learning tools in the area of neuroscience. Mathematically, in a 2D field, a linearly separable features vector can be separated by a line. A line equation is defined by *y* = *ax*+*b*. By replacing *x* with *x*_*1*_ and *y* with *x*_*2*_, the equation will become *a*(*x*1−*x*2)+*b* = 0. If we stipulate *x* = (*x*1,*x*2) and *w* = (*a*−1), we get *w*.*x*+*b* = 0, which gives the hyperplane equation. The hyperplane equation with a linearly separable output has the following form:


(14) $$f\,\left(y\right)=z^{T\,\phi }.\left(y\right)+b$$

where y represents the input data, *z_T_* represent a hyperplane, and ϕ(*y*) represents a function that map vector y into a high dimension. The elements z and b are appropriately scaled by the equal value, and the selected hyperplane in equation (14) remains stable. Furthermore, hyperplane can be making an exclusive pair of (z,b), which is represented by below formulation:


(15) $$\min|z^{T\,\phi }.\left(y_{i}\right)+b|=1,i=1,\ldots ,N,$$

where *y*_1_,*y*_2_,…,*y*_*N*_ represent the training vector. The hyperplane in equation (15) are recognized as canonical hyperplanes. The hyperplane is represented as below:


$$z^{T\,\phi }.\left(x\right)+b=0,w\,h\,i\,c\,h\,i\,s\,s\,a\,m\,e\,a\,s$$

(16) $$z^{T\,\phi }.\left(y\right)=0\,\left(w\,h\,i\,c\,h\,h\,a\,s\,m\,o\,r\,e\,d\,i\,m\,e\,n\,s\,i\,o\,n\,s\right)$$

For a feature *x* that does not fit the obtained hyperplane, the equation below represents it (Cortes and Vapnik, 1995):


(17) $$z^{T\,\phi }.\left(x\right)+b=\pm s\,||z||$$

where *s* is the measure of vector *x* to the defined hyperplane. Therefore, the output vector *fy* from SVM is exactly equivalent to the distance *sx* and z vector for the obtained hyperplane. Furthermore, in this study, we have utilized the kernel-support vector method, which is good to deal with the non-linear issue with the help of the linear classification method and which engages in swapping of the linearly un-classifiable vector into linearly classifiable. The concept inside this idea is a linearly unclassifiable vector that might be linearly classifiable in high dimensions. The kernel is mathematically defined as,


(18) $$K\,\left(x,y\right)={\left(x,y\right)}^{d}$$

where *x* and *y* represent features in the input and *d* represents the kernel element. Gaussian radial bias functions are represented by:


(19) $$K\,\left(x,y\right)=\exp\left(-\frac{{||x-y||}^{2}}{2\,\sigma^{2}}\right)$$

where *x* and *y* represent two samples input, which are vectors in input ||*x*−*y*||^2^ that can be represented as Euclidean distance in the square form among two features. σ represents kernel elements. Sigmoid function derived from the neural networks was used for activation, and the bipolar sigmoid function is utilized often for an artificial neuron, which is represented by


(20) $$K\,\left(x,y\right)=\tanh\;\left(\propto x^{T}\,y+c\right)$$

where *x* and *y* represent features in the input and ∝,*c* represents the kernel elements.

**FIGURE 3:**
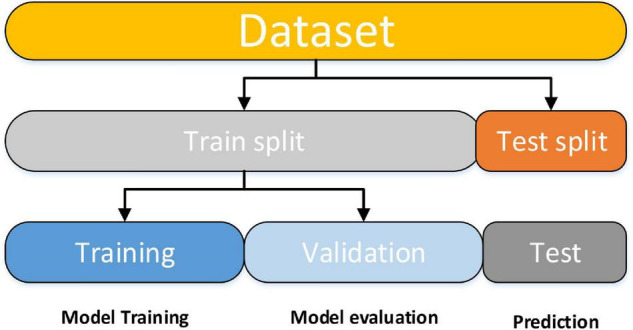
The data splitting stage is depicted in a diagram.

The SVM classifier was implemented by utilizing the Scikit-learn library (Pedregosa et al., 2011). The Scikit-learn library internally utilized LIBSVM (Chang and Lin, 2011) to handle all calculations. To obtain optimal classification accuracy, hyperparameters; cost *c* and γ (kernel width) of SVM must be tuned. With the aid of grid search and a 10-fold CV, these tuned optimal hypermeter values are automatically selected from the specified range of c = 1 to 9 and γ=1*e*^−4^ to 1. CV is a popular data shuffling and resampling technique for evaluating the generalization idea for the design of a predictive model and for preventing the underfitting or overfitting of the classifiers. CV is widely utilized in predictive modalities such as classification problems. In such types of issue, a framework is fitted with a known dataset, which is known as the training set, and a set of unknown samples against that model is evaluated, as the test set. The purpose is to have a testing sample for the model in the training stage and to then demonstrate how the process adopts various unknown datasets. Each phase of the CV engages the partition of the data samples into independent datasets, followed by an analysis of an individual sample. Subsequently, the study is validated on new independent subsets called testing samples. To lessen variability, numerous phases of CV are carried out by several partitions, after which an average of the results is considered. CV is a powerful procedure for evaluating model performance. Moreover, the data split features were applied in our model. The training data is used to train the machine learning (ML) classifier for subject group prediction across the provided features. After that, the classifier will be fine-tuned and tested on holdout data. To begin, model training entails a procedure in which ML passes the trained data *via* a process in which the classifier uncovers the train data patterns. As a result, the parameters are passed through to the target variables. As stated, our goal was to develop an ML classifier for the specific purpose of accurately identifying patients with AD and HC. We used supervised and ensemble learning models to propose an efficient ML classifier in the classification of subjects with AD to predict the AD patient status given a collection of independent variables. For crossvalidation purposes, we partitioned the dataset into three subgroups using this procedure. One set (test data) is used to forecast model performance, while the other two sets (training and validation) are used to evaluate model performance by training against new data. We randomly divided the entire dataset into a 70:30 ratio after data preparation, with 70% utilized for training and 30% used for testing. This will allow the system to generate fresh combinations each time the model is run, allowing for the most accurate prediction. The training dataset was divided into two subsets for training and validation after model training. Figure 3 is a brief explanation of each model.

In this study, accuracy, specificity, sensitivity, F1-score, and Receiver Operating Characteristic (ROC) curves were used for performing validation of the classifiers. We also calculated Cohen’s kappa values for each class group, which represent interrater reliability between two classes (Cohen, 1960). Kappa calculates the proportion of information scores in a table’s principal diagonal and then adjusts them for the amount of agreement that might be expected by chance alone. For two raters, the formula is $K=\frac{p_{0}-p_{e}}{1-p_{e}}$, where is the relative observed agreement between raters and is the hypothetical probability of chance agreement. In this method, we referred to HC as negative samples, patients with AD as positive samples, TN represents the number of negative sample sets that are correctly classified, total positive (TP) denotes the number of positive samples correctly categorized, false positive (FP) denotes the portion of negative dataset classified as positive, and false-negative (FN) denotes the number of positive datasets classified as negative samples. The accuracy, specificity, precision, and area under the curve are defined as follows:


(21) $$a\,c\,c\,u\,r\,a\,c\,y=\frac{T\,P+T\,N}{T\,P+F\,P+F\,N+T\,N}$$

(22) $$s\,p\,e\,c\,i\,f\,i\,c\,i\,t\,y=\frac{T\,N}{T\,N+F\,P}$$

(23) $$s\,e\,n\,s\,i\,t\,i\,v\,i\,t\,y=\frac{T\,P}{T\,P+F\,N}$$

The ROC curve, which is a curve obtained by plotting the TP rate versus the FP rate, can calculate the diagnostic capability of a binary classifier. The area under the ROC curve is proportional to the classifier performance.

## Results

### Findings From a Demographic and Clinical Approach

In AD over HC, AD over MCI, MCI over HC, and MCIs over MCIc, there was no significant age difference between groups. In all group combinations, however, there was a significant difference in MMSE (*P* > 0.05) and CDR (*P* > 0.05). AD has a male-predominant gender percentage while HC has a female-predominant gender percentage, whereas MCIs and MCIc have a female- and male-predominance percentage, respectively. Male dominance in AD is 58.73%, while female dominance in HC is 51.47%; whereas female and male dominance in MCIs and MCIc is 59.45 and 60%, respectively. These variables are described and analyzed in-depth in Table 1.

### Highly Sensitive Brain Network Features

**FIGURE 4:**
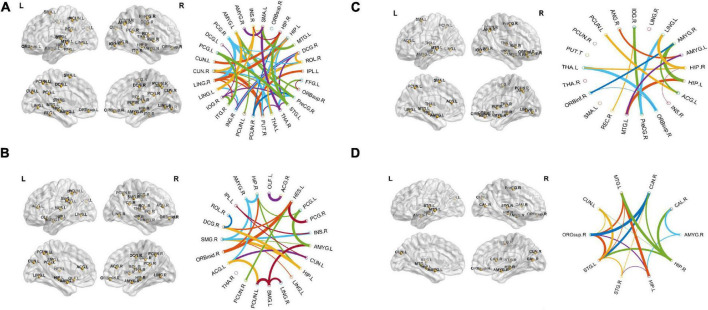
The location and networks of cortical regions with the highly discriminative attribute of top brain regions (BrainNet Viewer)^8^ and their corresponding circular connectivity (circularGraph) (http://www.mathworks.com/matlabcentral/fileexchange/48576-circulargraph): **(A)** Alzheimer’s disease (AD) vs. healthy control (HC) group, **(B)** AD vs. mild cognitive impairment (MCI) group, **(C)** HC vs. MCI group, **(D)** stable MCI (MCIs) vs. converted MCI (MCIc) group.

This section presents the top brain network features obtained by the JMI algorithm. Details about the selected network feature number and location of the AAL brain regions with their connectivity in the circular graph are presented in Figure 4 below. The feature reduction using the JMI method preserves all of the following attributes: betweenness centrality (BC), nodal path length (NL), and nodal degree (ND) features. We noted that, for all group classification NL trait contributed more as compared to the other two network features. The features selected show roughly similar features for the AD vs. HC and the MCIs vs. MCIc group and include the right precentral gyrus (PreCG.R), the left middle temporal gyrus (MTG.L), the left superior temporal gyrus (STG.L), the hippocampus (HIP.L and R), the amygdala left (AMYG.L), and the cuneal cortex right (CUN.R). For AD vs. MCI classification, the hippocampus (HIP.L and R), the cuneal cortex left (CUN.L), and the amygdala were selected along with other brain regions; similarly, for the HC vs. MCI group classification, the left middle temporal gyrus (MTG.L), the hippocampus (HIP.L and R), and the amygdala (AMYG.L and R) were selected along with other brain regions as shown in Figure 4. From this, we can conclude that the most affected brain region for all group classification analysis was mainly located on the middle temporal gyrus, the hippocampus, and the amygdala area followed by other brain regions. The location and brain region of these brain features are presented in Supplementary Tables 1–4.

### Voxel-Based Sensitive Features

**FIGURE 5:**
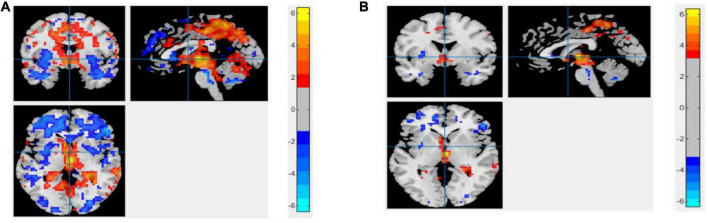
An univariate statistical two-sample test on region of homogeneity (ReHo) voxel maps among two classification groups **(A)** AD vs. HC **(B)** MCIs vs. MCIc. The threshold value was set to *p* < 0.05. The hot and cold bar represent negative and positive changes.

**FIGURE 6:**
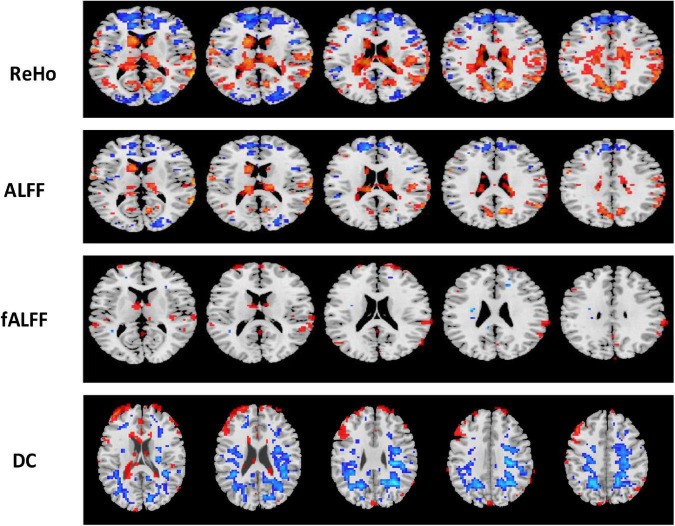
Univariate *t*-test difference maps between two classification groups, AD vs. HC, of four voxel maps.

**FIGURE 7:**
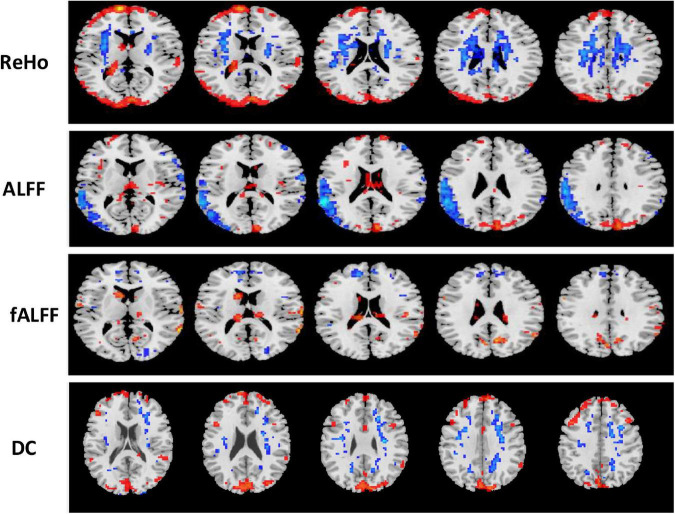
Univariate *t*-test difference maps between two classification groups MCIs vs. MCIc, of four voxel maps.

The region showing significant alteration in a univariate *t*-test are vital in achieving highly accurate differential prediction of the AD, MCI, and HC groups. For the optimal classification accuracy, previous studies (Arbabshirani et al., 2017) used the univariate statistical *t*-test to compute the group difference in the voxel-based analysis in the machine learning method. The main outcomes of the calculation are relied on statistical tests generally represented by *p*-values. Afterward, the excellent *p*-value only preserves the effective brain regions. Using the *t*-test of different group analyses, we created an analytically significant mask that preserved only the significant voxels difference between the two groups at *p* < 0.05 for ReHo, ALFF, and fALFF obtained from rs-fMR images. Consequently, adjusted individual voxel *p*-values of.05 were determined. Afterward, for SN, we utilized a well-validated region of interest (ROI) that included 12 posterior and 7 anterior SN nodes accessible at^9^ and were extracted using independent component analysis (Shirer et al., 2012), as presented in Supplementary Figure 5 and Supplementary Table 5. Similarly, to do a full-brain study of the areas impacted by AD and MCI, we utilized a frequently used graph-based metric of network architecture called degree centrality (DC). Individual network centrality maps were created in a voxel-by-voxel manner within the study mask. First, a voxel-based whole-brain correlation analysis was performed on the preprocessed functional runs. A correlation matrix was created by correlating the time course of each voxel inside the gray matter mask from each participant with the time course of every other voxel. The DC was calculated as the sum of the weights of the significant weighted connections for each voxel using an undirected adjacency matrix created by thresholding the correlation at *r* > 0.25 (Zuo et al., 2012). Finally, the DC on a voxel-by-voxel basis is calculated at the individual level. The highly significant brain regions obtained on the voxel-based analysis for the proposed method are shown in Figures 5–7 and in Supplementary Figures 6, 7. We have also presented the information of peak regions and their corresponding MNI co-ordinate in Supplementary Tables 6, 9. The most discriminative patterns obtained on the process and all information from regional coherence measures were calculated. This can suggest that various parts of the brain region go through various functional alterations because of MCI and Alzheimer’s. Therefore, to accomplish optimal diagnostic accuracy of the classification framework, it should cover the complementary information about altered brain patterns. One major discovery of this framework is that the effective and important regional voxel-based features can complement the brain network features obtained from the rs-fMRI data and structural features from sMRI.

### Classification Results

**FIGURE 8:**
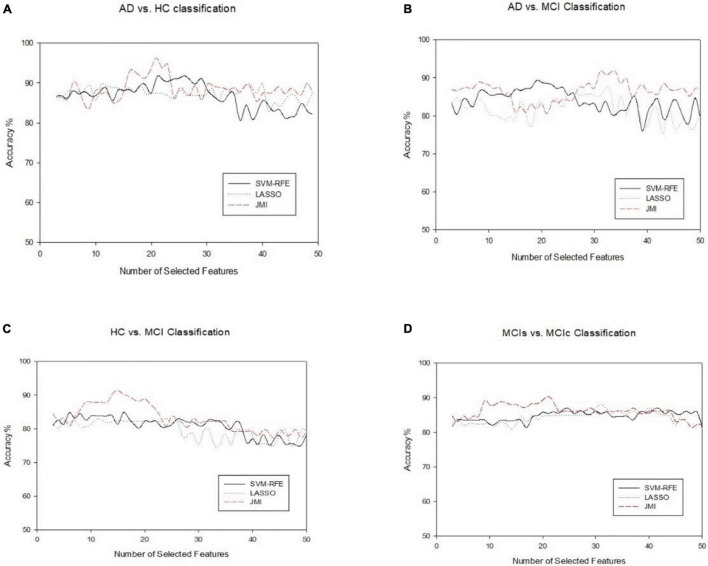
An illustration of the performance of three feature selection algorithms for classifying four different groups on combined features vectors **(A)** AD vs. HC, **(B)** AD vs. MCI, **(C)** HC vs. MCI, and **(D)** MCIs vs. MCIc. The *x*-axis represents the number of selected features, while the *y*-axis represents the classification accuracy.

In this section, we evaluated the diagnostic performance of the purposed method on sMRI of the hippocampus, the amygdala features along with brain network features, and the voxel-based features of rs-fMRI and have combined all of them with that of SVM and RF classifiers for respective binary classification. We implemented and analyzed the three features obtained from sMRI and rs-fMRI images separately as well as combined. We used the hippocampus and the amygdala volume, as well as voxel features, as complementary features for the brain network, which help to improve the classification accuracy for AD and MCI diagnosis. The combined procedure shows great potential for the classification task with higher AUC, accuracy, better sensitivity, and precision. For brain network features, we measured the different features such as ND, NL, and BC. Through a series of threshold values in the cost, 5–25% of best and stable results were utilized for diagnostic classification of each group, which is presented in Supplementary Figures 1–4. Similarly, for voxel-based features, we calculated the five voxel features, namely ReHo, ALFF, fALFF, DC, and SN: group difference univariate *t*-test. We evaluated our feature reduction and classification algorithm on feature vectors using a 10-fold CV. First, we partition the data into 10 equal-sized subsets (folds) which contain 90% subset for training and the remaining 10% of the test subjects. Then, features selection was carried out on the training subsets. We implemented the distinct features selection algorithm to select the important feature sets to optimize the classifier performance. Based on the obtained top selected features set, SVM and RF classifiers were trained. Because each feature had a distinct scale, we linearly ascended each training feature to simulate to a range between 0 and 1 in our case; the same scaling procedure was then used on the test dataset. In our scenario, the RBF kernel outperforms other kernels because of the modest number of features used. For each test and training subset, we implemented independent feature selection to escape the feature selection bias amid a 10-fold CV. We measured cross-validated accuracy for classifiers on a given number of feature sets and plotted the numbers of selected features against the accuracy as shown in Figure 8 for each group classification.

**FIGURE 9:**
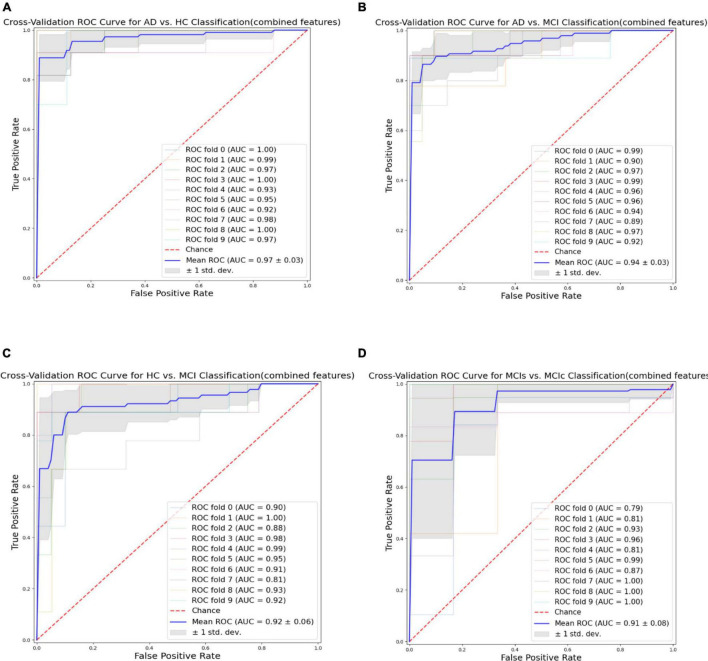
Receiver operating characteristics (ROC) curve for **(A)** the AD vs. HC group, **(B)** the AD vs. MCI group, **(C)** the HC vs. MCI group, and **(D)** the MCIs vs. MCIc group for combined features vectors: Hippocampus, amygdala, brain network (BN), and voxel (Combined).

**FIGURE 10:**
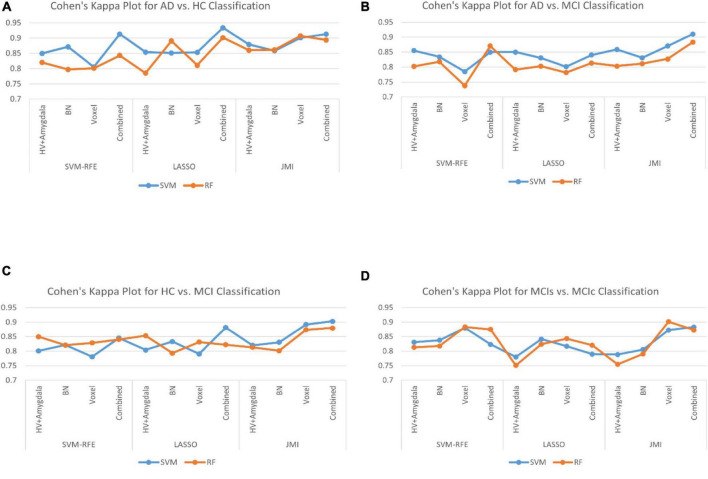
The Cohen’s Kappa index for **(A)** AD vs. HC group, **(B)** AD vs. MCI group, **(C)** HC vs. MCI group, and **(D)** MCIs vs. MCIc group for individual and combined feature set and different feature selection algorithm.

Finally, we evaluated the AUC as shown in Figure 9, and accuracy, sensitivity, specificity, F1-score, and Cohen’s Kappa in Figure 10 for individual and combined feature set and different features selection algorithm as presented in Tables 2–5. Table 2 presents the classification of AD against HC. In this study, we compared the performance of different features selection methods on the different feature sets with SVM and RF classifiers. The Joint Mutual Information (JMI) feature reduction technique with SVM classifiers outperforms all other techniques contemplated with the highest AUC and accuracy. For AD vs. HC diagnostic classification, the integrated (Hippocampal + Amygdala + BN + Voxel) feature vectors performed well in comparison with individual feature set with 97.03% AUC, 95.87% accuracy, 97.35% sensitivity, and 95.95% specificity along with 96.33% F1-score and.913 Cohen’s Kappa index, respectively. We did not notice the significant classification difference between the SVM-RFE, and LASSO features selection algorithm for AD vs. HC. In comparison with the RF classifier, there was 1–6% better performance in terms of accuracy with SVM for AD vs. HC classification on different features vectors, but we noticed slightly better specificity for combined features with LASSO for RF classifier. For voxel features with JMI feature selection, we noticed the same pattern for RF classifiers that perform equally with SVM classifiers in terms of accuracy. For hippocampal and amygdala volume, RF classifiers with SVM-RFE features selection show good performance in terms of F1-score. Similarly, for the classification performance of AD vs. MCI as shown in Table 3, we obtained the 94.03% AUC, 92.45% accuracy, 95.98% sensitivity along with 90.45% specificity, 93.75% F1-score, and.9105 of Cohen’s Kappa index, respectively. For HC vs. MCI classification as shown in Table 4, we achieved the highest classification accuracy on the JMI feature selection method. Although there was not much difference between the individual features method, JMI significantly improved for the combination of the hippocampal, amygdala, brain network, and voxel-based features sets with 92.06% AUC, 90.35% accuracy, and 94.34% sensitivity along with 92.11% specificity, 94.13% F1-score, and.9035 of Cohen’s Kappa score. More importantly, from the results presented in Tables 2–5, brain network features perform well as compared to the hippocampal, amygdala (sMRI), and voxel-based (rs-fMRI) features for individual features in most of the cases.

**TABLE 2:** A 10-fold cross-validated binary classification performance for Alzheimer’s disease (AD) vs. healthy control (HC) groups using support vector machine (SVM) and Random Forest (RF) classifiers.

Performance Matrix												
		SVM						RF				
Feature selection method	Features	AUC	ACC	SEN	SPE	F1	Cohen’s Kappa	ACC	SEN	SPE	F1	Cohen’s Kappa
SVM-RFE	HV + Amygdala	87.12	84.51	91.13	85.07	85.45	0.8501	78.03	86.12	86.33	87.13	0.8201
	BN	90.42	90.13	81.04	85.12	84.13	0.8713	88.47	87.35	90.07	82.15	0.7972
	Voxel	92.17	88.97	90.71	95.09	88.46	0.8055	84.78	92.01	86.42	84.13	0.8013
	Combined	93.14	92.98	95.03	92.71	83.78	0.9131	91.05	91.23	88.88	82.23	0.8430
LASSO	HV + Amygdala	85.99	82.75	82.03	87.59	82.20	0.8538	80.05	75.43	83.11	84.05	0.7851
	BN	90.07	88.95	95.04	84.45	85.77	0.8512	87.74	86.71	91.08	87.71	0.8903
	Voxel	91.75	88.13	92.14	93.25	90.65	0.8531	87.31	90.45	87.72	76.11	0.8113
	Combined	95.95	94.13	93.53	95.51	84.38	0.9345	90.46	93.04	84.22	84.11	0.9012
JMI	HV + Amygdala	89.45	85.33	89.05	84.83	86.15	0.8791	80.85	87.59	82.2	85.38	0.8601
	BN	93.95	92.75	93.03	89.08	92.45	0.8583	87.75	87.47	90.35	91.37	0.8614
	Voxel	94.41	92.46	95.43	88.81	91.15	0.9010	92.51	95.00	85.87	93.15	0.9071
	Combined	97.03	95.87	97.35	95.95	96.33	0.9130	93.23	95.31	94.93	92.37	0.8935

**TABLE 3:** A 10-fold cross-validated binary classification performance for AD vs. mild cognitive impairment (MCI) groups using SVM and RF classifiers.

Performance Matrix												
		SVM						RF				
Feature selection method	Features	AUC	ACC	SEN	SPE	F1	Cohen’s Kappa	ACC	SEN	SPE	F1	Cohen’s Kappa
SVM-RFE	HV + Amygdala	85.27	78.14	87.91	84.73	82.75	0.855	77.45	83.18	84.17	84.18	0.8021
	BN	91.42	84.85	95.12	87.45	88.13	0.8342	80.25	90.43	84.56	77.45	0.8178
	Voxel	85.32	82.83	84.51	93.13	83.52	0.7847	81.75	92.53	84.98	81.78	0.7380
	Combined	93.71	87.18	92.45	90.14	91.75	0.8501	86.45	88.37	84.73	82.51	0.8703
LASSO	HV + Amygdala	84.9	80.83	93.11	87.04	84.19	0.8503	78.51	82.58	78.95	84.45	0.7913
	BN	90.34	88.74	94.45	90.87	87.14	0.8305	85.91	90.98	87.42	83.31	0.8033
	Voxel	86.73	85.24	95.01	88.11	85.46	0.8013	85.41	91.21	84.63	78.98	0.7818
	Combined	93.91	90.45	94.46	91.71	89.58	0.8401	87.75	92.13	90.03	85.54	0.8135
JMI	HV + Amygdala	82.75	81.27	87.72	81.27	88.35	0.8587	79.31	83.24	81.18	85.03	0.8035
	BN	91.73	91.32	94.43	87.17	88.75	0.8309	86.08	87.53	88.14	84.23	0.8118
	Voxel	92.08	88.01	93.13	89.57	91.33	0.8703	87.89	93.58	89.13	85.85	0.8273
	Combined	94.03	92.45	95.98	90.45	93.75	0.9105	90.75	95.15	87.73	91.03	0.8831

**TABLE 4:** A 10-fold cross-validated binary classification performance for HC vs. MCI using SVM and RF classifiers.

Performance Matrix													
		SVM							RF				
Feature selection method	Features	AUC	ACC	SEN	SPE	F1	Cohen’s Kappa	ACC		SEN	SPE	F1	Cohen’s Kappa
SVM-RFE	HV + Amygdala	82.85	77.32	90.54	84.13	84.71	0.8010	76.53		86.15	90.23	78.97	0.8501
	BN	86.01	85.42	84.12	81.47	84.13	0.8210	78.56		84.45	88.11	78.47	0.8203
	Voxel	87.31	86.53	93.12	87.89	85.45	0.7809	84.01		90.35	88.75	80.10	0.8283
	Combined	91.21	85.21	93.45	92.11	88.79	0.8451	84.36		87.84	85.01	79.45	0.8401
LASSO	HV + Amygdala	84.92	76.95	87.3	82.08	82.83	0.8045	75.03		85.91	90.07	87.18	0.8531
	BN	87.42	82.15	88.63	87.45	84.88	0.8331	81.52		87.38	82.88	85.45	0.7933
	Voxel	87.13	80.45	86.85	91.12	86.91	0.7903	78.96		88.45	86.23	76.14	0.8311
	Combined	90.03	84.74	85.77	91.03	90.11	0.8815	82.41		87.15	82.95	80.75	0.8220
JMI	HV + Amygdala	83.57	79.45	88.45	79.85	83.50	0.8201	77.13		78.95	85.52	85.15	0.8130
	BN	91.83	86.15	92.09	94.15	87.71	0.8305	83.87		91.54	84.35	81.33	0.8015
	Voxel	90.57	86.43	85.15	86.01	91.23	0.8917	84.25		88.32	83.03	89.77	0.8738
	Combined	92.06	90.35	94.34	92.11	94.13	0.9035	85.15		91.12	85.31	84.95	0.8805

**TABLE 5:** A 10-fold cross-validated binary classification performance for stable MCI (MCIs) vs. converted MCI (MCIc) groups using SVM and RF classifiers.

Performance Matrix												
		SVM						RF				
Feature selection method	Features	AUC	ACC	SEN	SPE	F1	Cohen’s Kappa	ACC	SEN	SPE	F1	Cohen’s Kappa
SVM-RFE	HV + Amygdala	83.35	76.43	82.75	78.41	82.14	0.8310	75.02	80.37	85.3	79.41	0.8129
	BN	88.32	84.46	90.11	85.27	90.17	0.8375	80.45	87.52	91.15	80.14	0.8175
	Voxel	84.54	83.25	91.95	88.11	83.52	0.8805	80.34	86.33	80.17	84.75	0.8835
	Combined	90.19	85.32	92.87	88.03	88.73	0.8231	82.13	84.85	87.85	87.02	0.8750
LASSO	HV + Amygdala	85.23	75.10	86.35	83.57	82.17	0.7803	74.91	74.74	81.50	77.13	0.7512
	BN	86.37	80.98	92.84	86.38	84.56	0.8415	76.89	84.77	80.11	77.91	0.8240
	Voxel	84.33	84.45	90.37	87.33	89.47	0.8170	82.31	86.13	84.71	79.79	0.8430
	Combined	89.75	85.11	92.37	90.15	85.12	0.7897	83.39	91.18	84.27	84.18	0.8203
JMI	HV + Amygdala	85.52	78.45	88.5	84.05	83.14	0.7888	77.54	79.57	82.35	79.09	0.7545
	BN	88.73	84.38	94.17	91.13	90.03	0.8055	82.45	87.31	81.41	90.93	0.7905
	Voxel	87.97	85.14	92.77	87.53	91.11	0.8730	84.24	90.34	82.59	88.15	0.9015
	Combined	91.08	88.03	94.85	89.71	93.17	0.8831	85.31	92.17	87.15	90.57	0.8733

*AUC, area under curve; ACC, accuracy; SEN, sensitivity; SPE, specificity; F1, F-score; SVM, support vector machine; RF, random forest; SVM-RFE, SVM recursive feature elimination; LASSO, least absolute shrinkage and selection operation; JMI, joint mutual information; HV, hippocampus volume; BN, brain networks).*

Similarly, the classification performance for the less commonly reported group of MCIs vs. MCIc using different features selection methods is listed in Table 5. Like the previous pattern, the highest diagnostic classification results in terms of AUC, accuracy, specificity, and sensitivity were calculated with the JMI feature selection method as compared to other features selection technique. However, for MCIs vs. MCIc classification, there was no significant difference between the performance of SVM-RFE and the LASSO feature selection as compared to other different group classifications as shown in Tables 2–5. For the MCIs vs. MCIc classification, we obtained the 91.08% AUC, 88.03% accuracy, 94.85% sensitivity, and 89.71% specificity along with 93.17% of F1-score, and.8831 of Cohen’s Kappa index, respectively, for combined feature set, i.e., hippocampal and amygdala features (sMRI) as well as BN and Voxel (rs-fMRI) features. From Table 5 for the MCIs vs. MCIc classification, SVM-RFE and LASSO feature selection method also shows the potential to compete the JMI feature selection with 90.19% and 89.75% AUC along with 85.32% and 85.11% accuracy, respectively. We also observed that accuracy is significantly increased while classifying MCIs vs. MCIs by integrating hippocampal and amygdala volume of sMRI along with brain networks and voxel features of rs-fMRI. Overall, we noticed that, as compared with RF classifiers, SVM classifiers perform better for almost all feature selection techniques and all three different kinds of feature sets. This may be due to lesser training data than the number of feature set. SVM is better than RF when there were a large number of features and lesser training data and RF is better for multiclass problems, while SVM is better for binary classification.

From all these reported results, it has clear evidence that the utilization of JMI as features selection algorithm for MCI and AD against HC classification and conversion prediction of MCI shows the great potentiality using SVM classifiers with a combination of structural features (hippocampal and amygdala), brain networks, and voxel-based (rs-fMRI) features. More importantly, the AUC curve as illustrated in Figure 9 below for all classification groups shows that the proposed model was quite stable.

## Discussion

Alzheimer’s disease can be detected earlier, which can aid with therapy and avoid brain tissue damage. For the diagnosis of AD, researchers have used a variety of statistical and machine learning models. In clinical research, the MMSE score, MRI analysis (normalized whole-brain volume, hippocampal volume), biomarkers based on CSF, such as amyloid–42, and combined biomarkers have shown a great potentiality for AD diagnosis (van Maurik et al., 2017). The closeness between AD MRI data and normal healthy MRI data of older persons makes detection of AD difficult. A recent study suggested that decreased hippocampus subfield volumes have been commonly observed in dementia disorders such as AD and dementia with Lewy bodies (DLB) (Delli Pizzi et al., 2016; Mak et al., 2016, 2017). The volumes of the CA1, CA2-3, CA4, DG, and total subiculum (subiculum, presubiculum, and parasubiculum) are reduced in AD, according to one study (Mak et al., 2017). Similarly, the rs-fMRI data not only involve peculiar numerical features but also present rich dynamic temporal information. Several works of literature that relied on rs-fMR have been tested and verified for the diagnostic classification of MCI and Alzheimer’s from the healthy population. However, those previous studies either focus only on structural features, the graph theory approach, or the voxel-based approach and lose the full potentiality of combing structural features (hippocampal-amygdala) and brain network with voxel-based features. Therefore, to examine the full possibility of rs-fMRI and sMRI on AD diagnosis, we presented insights into the performance for diagnostic classification of all four binary classification groups by combining the hippocampus subfield and the amygdala nuclei volume from sMRI along with brain network and voxels-based features (ReHo, fALFF, ALFF, DC, and SN) from rs-fMRI. Besides this, we also proposed the different feature selection algorithms for the classification of AD vs. HC, AD vs. MCI, HC vs. MCI, and the less commonly reported group, MCIs vs. MCIc. Our experimental outcome indicated that each feature set is important to achieving good classification performance.

**FIGURE 11:**
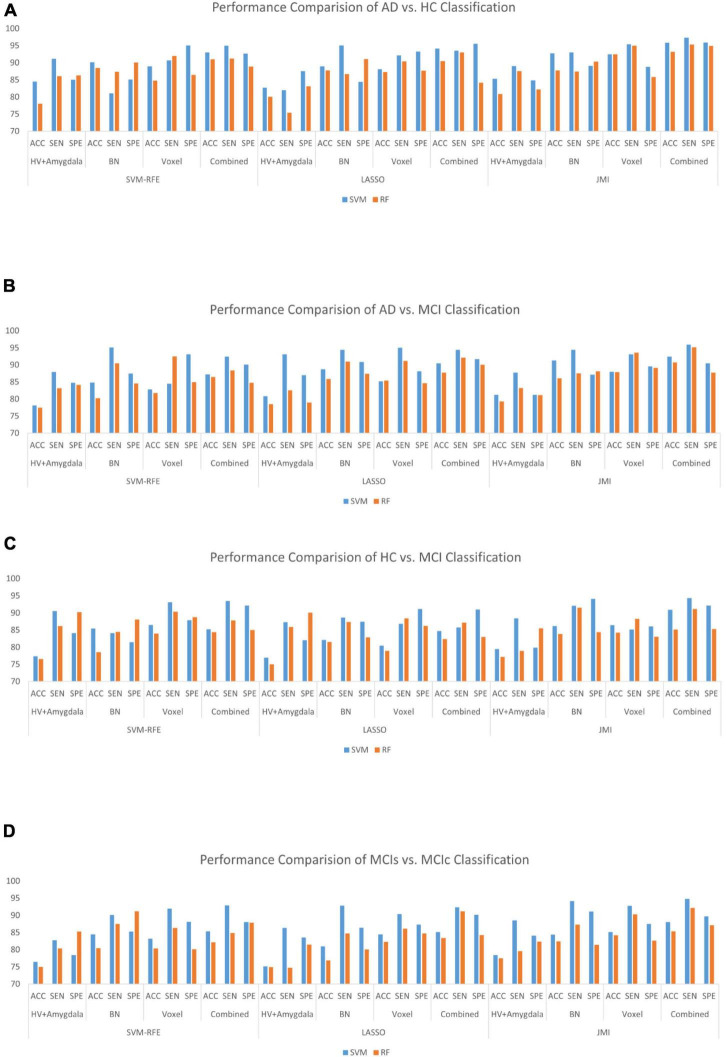
Bar graph with different features selection methods **(A)** the AD vs. HC group, **(B)** the AD vs. MCI group, **(C)** the HC vs. MCI group, and **(D)** the MCIs vs. MCIc group on different feature vectors: Hippocampal-amygdala volume, Brain Network (BN), Voxel, and combined features set.

Several recent works of literature have analyzed the neuroimaging method for discriminative classification of AD, with the target on patients with MCI, who may or may not convert to Alzheimer’s, and identifying an individual with Alzheimer’s from HC. However, it is hard to do a direct comparison with the existing state-of-art methods due to the majority of works of literature utilizing different datasets and classification methods, which both significantly affected the performance accuracy. With the combination of different feature selections with different classifiers for AD vs. HC and MCIs vs. MCIc, the binary classification of previous works of literature has reported the accuracy of different ranges as shown in Tables 6, 7. These works of literature utilized the ADNI database to evaluate their proposed method, and we can clearly see that the classification accuracy was influenced by the number of subjects, and the accuracy decreased as the number of subjects increased. As reported in the Results section, the highest classification accuracy for AD vs. HC and MCIs vs. MCIc obtained in this study is 95.87 and 88.03%, using the combination of the features with JMI feature selection, which is visualized in Figure 11. If we compare the obtained results for AD vs. HC and MCIs vs. MCIc classification; our framework outperforms the current state-of-art method. The majority of the studies, including Khazaee et al. (2015) and Lama and Kwon (2021), have utilized a limited number of datasets because of the limited number of fMRI data availability in the ADNI data bank. For MCIs vs. MCIc, the accuracy of prior approaches (Khazaee et al., 2015; Zhang et al., 2021) for constructing brain networks was lower than that of the current study since they solely analyzed functional aspects. Only Hojjati et al. (2017) classified diseases using the rs-fMRI graph theory and the machine learning technique (mRMR, FS) with a 91.4% classification accuracy. However, the sample size (of only 18 people) was insufficient, and the result was not generally representative. In this study, we used rs-fMRI and sMRI features to perform binary classifications and found that combining structural and functional MRI data improved classification performance. In our proposed framework, we found that combined sMRI (hippocampal subfield and amygdala nuclei volume) and rs-fMRI (brain networks and voxel) outperformed a single sMRI or rs-fMRI model for two-group classifications (MCIs and MCIc) with improvement in accuracy (Ardekani et al., 2017; Zhang et al., 2021) as shown in Table 7. As a result, in the current work, we proposed to combine sMRI and rs-fMRI for disease classification. Schouten et al. used sMRI and fMRI to differentiate 16 patients with AD from 22 normal controls. They discovered that integrating features from two modalities increases classification performance, and they attained an accuracy of 89.5% in AD vs. HC classification (Schouten et al., 2016). All the state-of-art studies presented here analyzed and performed the classification task and made a conclusion. Furthermore, we also carried out our proposed method using ADNI dataset with a larger number of individuals as compared to existing works of literature with cross-validation. It was challenging to identify MCIc subjects since we utilized baseline sMRI and rs-fMRI images in these participants and they transitioned to AD 6 to 36 months later. They exhibited heterogeneity in their conversion time to AD, ranging from 6 to 36 months. Patients with MCIc who converted to AD over a longer period of time (e.g., 36 months) may have a comparable brain network and structure at baseline as compared to patients with MCIs who did not convert to AD. The brain networks and structures of patients with MCIc who progressed to AD in a shorter period (e.g., at 6 months) may on the other hand be comparable to those of patients with AD. In addition, individuals with MCIc were the only unstable group of patients that progressed from MCI to AD over the 36-month follow-up period. In fact, for at least 36 months, patients in the MCIs, AD, and HC groups remained stable and did not transition to another group. Furthermore, we noticed instability in patients with MCIc, as some of them converted to AD and then returned to MCI after 36 months. Unlike earlier research, our study examined not only the conversion sensitivity of the two groups of patients MCIs/MCIc but also analyzed the brain regions of other patient groups. The highly responsive brain regions selected from the two groups are listed in Figure 4 and Supplementary Tables 1–4. It is also worth mentioning that betweenness centrality contributed 70–75% of the features for brain networks and 30–35% for the feature combination. Our findings imply that the betweenness centrality in a functional network conveys more disease information and that the top selected features are more responsive to more efficient detection for MCIs vs. MCIc and HC vs. MCI. Our findings are consistent with earlier research, and these specific brain areas have been linked to AD and MCI conversion (Liu et al., 2013). The importance of numerous brain areas in MCI pathology has long been acknowledged.

**TABLE 6:** Performance comparison of AD vs. HC with state-of-the-art methods.

References	Cohort	Features	Classifier	Accuracy	AUC
de Vos et al., 2018	AD/HC(77/173)	FC matrices, FC dynamics, ALFF	Group Lasso LR	–	85%
Khazaee et al., 2015	AD/HC (34/45)	Graph measures	Naïve Bayes	93.30%	–
Lama and Kwon, 2021	AD/HC (31/31)	Brain Network	LSVM	90.63%	N/A
**Our method**	**AD/HC (33/35)**	**sMRI, Brain Network and Voxel features of rs-fMRI**	**SVM**	**96.95%**	**98**

*Bold value represents the results obtained form proposed method.*

**TABLE 7:** Performance comparison of MCIs vs. MCIc with state-of-the-art methods.

References	Cohort	Features	Classifier	Accuracy	AUC
Moradi et al., 2015	sMCI/pMCI	MRI, age and cognitive measure	LDS	82.72%	0.902
	(100/16)				
Ardekani et al., 2017	sMCI/pMCI (78/86)	Hippocampal volumetric (sMRI)	RF	82.30%	N/A
Hojjati et al., 2017	MCIc/MCInc (18/62)	rs-fMRI, graph theory	SVM	91.40%	N/A
Zhang et al., 2021	MCIc/MCInc (30/35)	rs-fMRI, sMRI, graph theory	SVM	84.71%	0.88
**Our method**	**MCIs/MCIc (30/31)**	**sMRI, Brain Network and Voxel features of rs-fMRI**	**SVM**	**87.78%**	**93.8**

*Bold value represents the results obtained form proposed method.*

Previous studies noted the network distortion in the temporal lobe area in individuals with Alzheimer’s (He et al., 2009). In other studies (Liu et al., 2013) also noted the functional loss in the middle temporal gyrus (MTG) and PreCG in AD patients. In comparison with previous literature, we notice that the temporal lobe area may be more damaged in the AD and initial MCI. The MTG was highly sensitive in the feature selection for MCI and AD classification. The nodal degree in the superior temporal gyrus (STG), Cuneus (CUN), precentral gyrus (PreCG), and MTG as well as the betweenness centrality in the hippocampus (HIP), Amygdala (AMYG), and inferior temporal gyrus (ITG) were shown to be discriminative in distinguishing AD from HC. Similar trends were followed by MCIs from MCIc classification. The nodal degree in the STG, middle temporal gyrus (MTG), and CUN as well as the betweenness centrality in the amygdala (AMYG) and Hippocampus (HIP) were shown to be discriminative in distinguishing MCIs from MCIc Supplementary Table 4. In summary, the highly sensitive features were selected for the brain network using the JMI algorithm. Moreover, the selected brain area carries more information about the disease with more sensitive features which leads to more accurate performance. The temporal region plays an important role in MCI and AD. We also suggested that the other region such as the caudate nucleus, superfrontal gyrus, orbitofrontal cortex, occipital, etc. regions for further exploration of the disease pathology in AD.

### Limitations

Our study has a few drawbacks. First, the sample size is limited, which may impair the robustness of the group’s statistical analysis. Further analysis with a bigger sample size with different datasets should be carried out. The unbalance data is another drawback. We aimed to analyze high-quality data with more balanced samples for feature selection and classification in the future or to design a more robust approach that enhances classification accuracy and generalization; the model’s generalization should be considered by using a different database besides ADNI. Because the ADNI database is growing, future research should acquire a bigger sample and should balance the number of individuals. Future research should also look at different networks analysis and classification approaches in different phases of AD, as well as the interpretability of functional brain abnormalities. The ability to evaluate the models’ resilience across numerous data sets will be required. We think that the follow-up data within the subject can better show the brain area where the sensitive features of the altered biomarker are present in terms of subject design. More significant and accurate findings may be produced if participants may record follow-up data through cognitive training while also maintaining a baseline control.

## Conclusion

Alzheimer’s disease is an irreversible and a leading health problem in older age; it is important to consider the protective response and to slow down the onset of the advancement of Alzheimer’s. Thus, the proper identification of various stages of Alzheimer’s and MCI progression is important. In this article, we utilized the hippocampal subfield and the amygdala nuclei volume obtained from sMRI in combination with brain network features and multi-measure features obtained from rs-fMRI. So far, several anatomical MRI imaging biomarkers for AD diagnosis have been identified. The use of the cortical and subcortical volume, the hippocampus, and the amygdala volume has proven to be beneficial in distinguishing patients with AD from the healthy population. Similarly, the rs-fMRI data provides specific numerical information but also contributes to the rich dynamic temporal correlation. However, those preceding studies used either of the biomarker hippocampal subfield and amygdala volume, brain networks, or voxel-based multi-measure features separately. Thus, to analyze the full potentiality of sMRI and rs-fMRI in AD identification, we utilized the combined features in our studies. Additionally, we utilized and compared the different features selection algorithm to select the optimal feature set to obtain the maximum classification accuracy. We also compared the performance of SVM with an RF classifier. From the results obtained, JMI feature selection with the SVM algorithm among all others significantly improved the performance accuracy.

## Data Availability Statement

Publicly available datasets were analyzed in this study. This data can be found here: the dataset used in this study were acquired from ADNI homepage, which is available freely for all researcher and scientist for experiments on Alzheimer’s disease and can be easily downloaded from ADNI websites: http://adni.loni.usc.edu/about/contact-us/. The raw data supporting the conclusions of this article will be made available by the authors, without undue reservation.

## Ethics Statement

The studies involving human participants were reviewed and approved by IRB no. is 2-1041055-AB-N-01-2019-45/2020-72 in Chosun University. The patients/participants provided their written informed consent to participate in this study.

## Author Contributions

UK developed the concept and handled the analysis. G-RK reviewed the concept and verified the results. Both authors reviewed and contributed to the manuscript and have approved the final version.

## Conflict of Interest

The authors declare that the research was conducted in the absence of any commercial or financial relationships that could be construed as a potential conflict of interest.

## Publisher’s Note

All claims expressed in this article are solely those of the authors and do not necessarily represent those of their affiliated organizations, or those of the publisher, the editors and the reviewers. Any product that may be evaluated in this article, or claim that may be made by its manufacturer, is not guaranteed or endorsed by the publisher.

## Footnotes

1
^†^ http://adni.loni.usc.edu/

2
http://surfer.nmr.mgh.harvard.edu/

3
http://www.fil.ion.ucl.ac.uk/spm

4
http://restfmri.net

5
https://surfer.nmr.mgh.harvard.edu/fswiki/HippocampalSubfieldsAndNucleiOf Amygdala

6
https://www.mathworks.com

7
https://github.com/dgleich/matlab_bgl

8
http://nitrc.org/projects/bnv/

9
http://findlab.stanford.edu/research.html
