# Supplementary material for: Alzheimer’s Disease Diagnosis and Biomarker Analysis Using Resting-State Functional MRI Functional Brain Network With Multi-Measures Features and Hippocampal Subfield and Amygdala Volume of Structural MRI
Source: Front Aging Neurosci. 2022 May 30;14:818871. doi: 10.3389/fnagi.2022.818871 (PMC9190953; doi:10.3389/fnagi.2022.818871)
Supplement: Supplementary file 1 [file Data_Sheet_1.pdf]

Supplementary Material

Alzheimer's Disease Diagnosis and Biomarker Analysis using rs-fMRI Functional Brain Network with Multi-measures Features and Hippocampus, Amygdala Sub-field of sMRI

1 Supplementary Figures and Tables

1.1 Supplementary Figures for Brain Networks

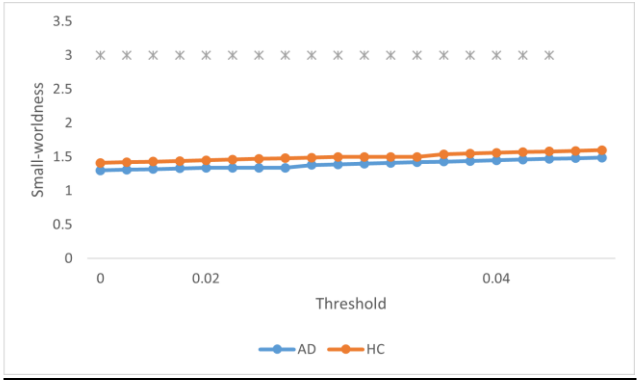

a)

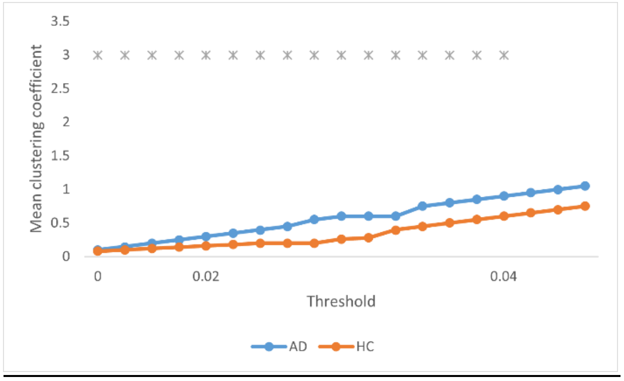

b)

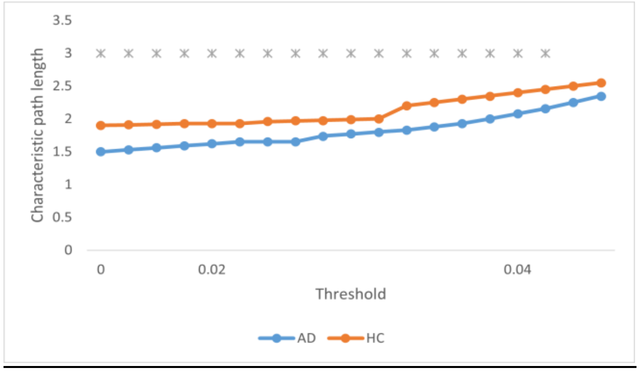

c)

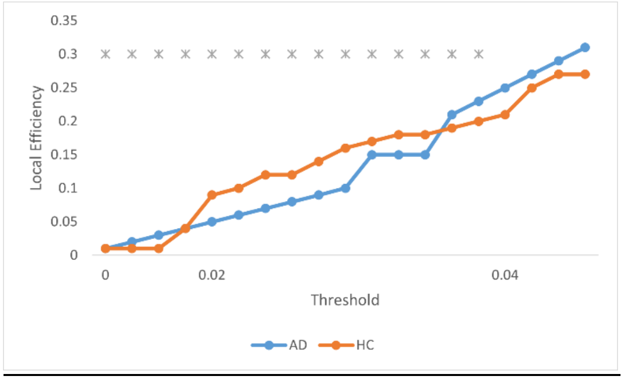

d)

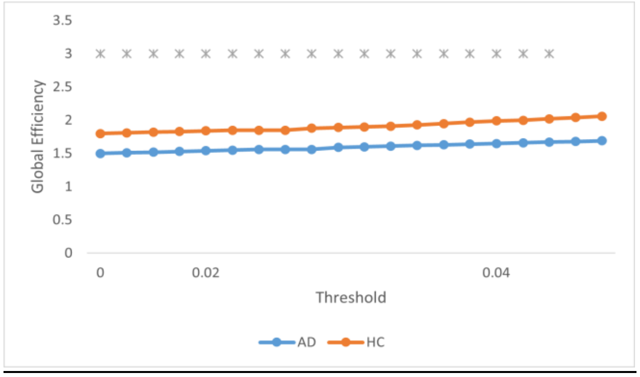

e)

Supplementary Figure 1. Group difference and network parameters comparison for AD vs. HC under different threshold value, where figure a) Small-worldness b) mean clustering coefficient c) characteristic path length d) local efficiency and e) global efficiency. * Represents the significant group level difference with two sample t-test at $P < 0.05$.

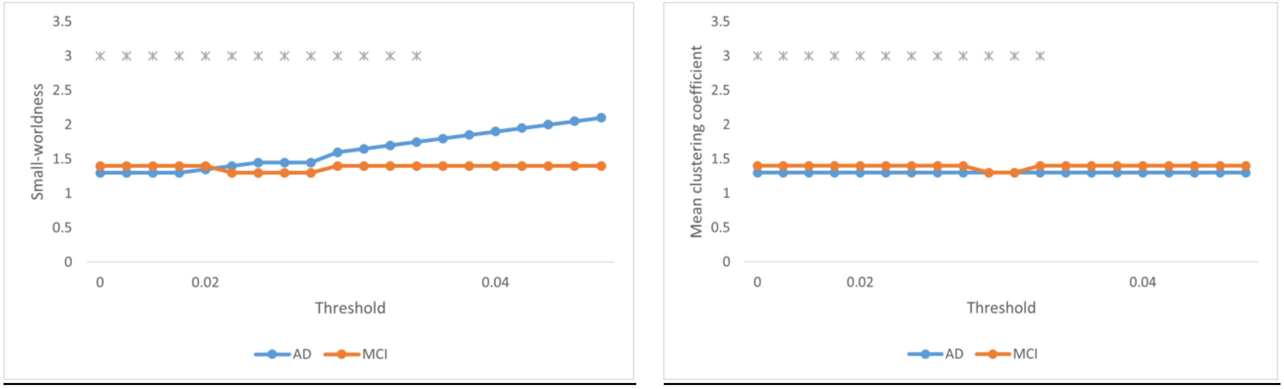

a) b)

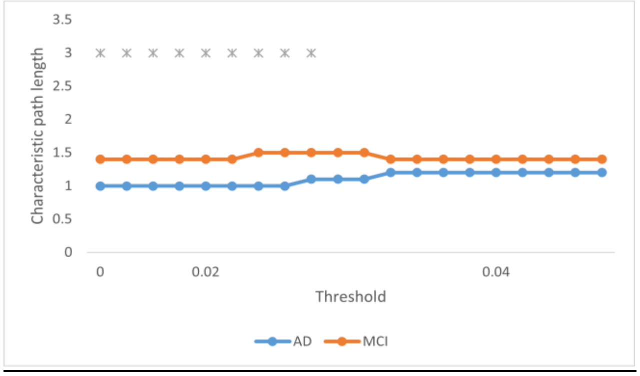

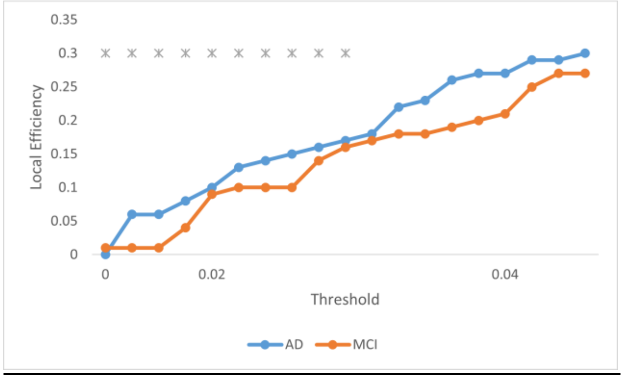

c)

d)

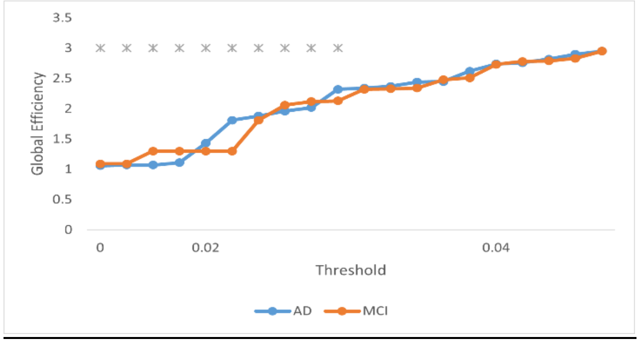

e)

Supplementary Figure 2. Group difference and network parameters comparison for AD vs. MCI under different threshold value, where figure a) Small-worldness b) mean clustering coefficient c) characteristic path length d) local efficiency and e) global efficiency. * Represents the significant group level difference with two sample t-test at P < 0.05.

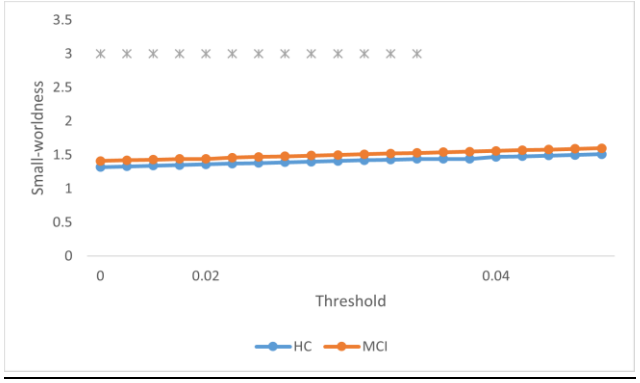

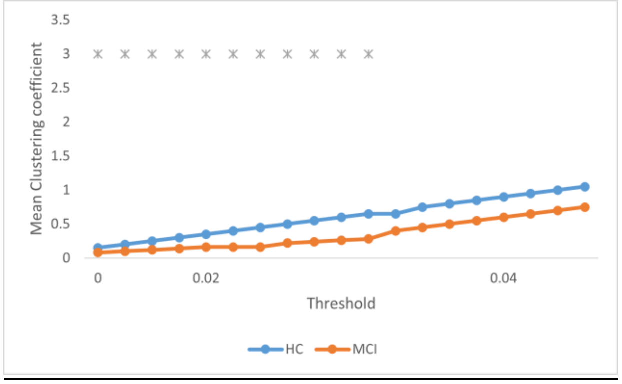

a)

b)

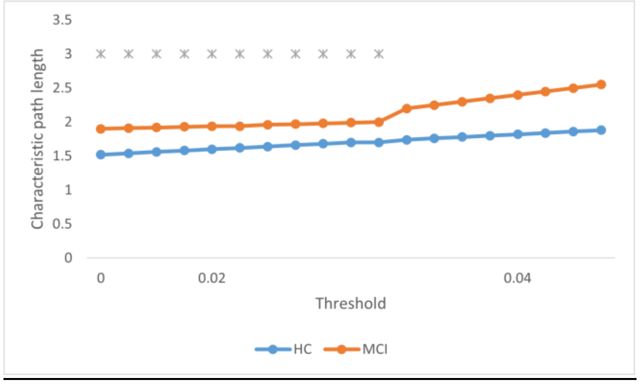

c)

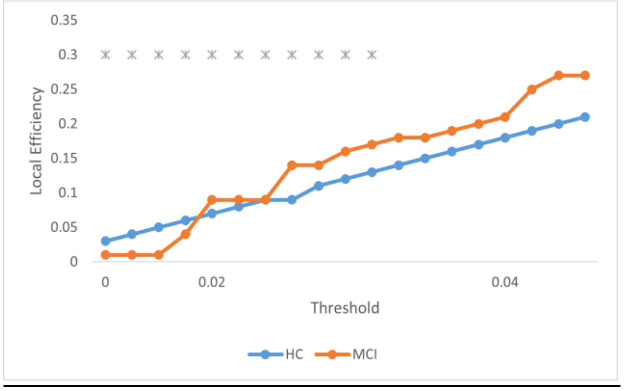

d)

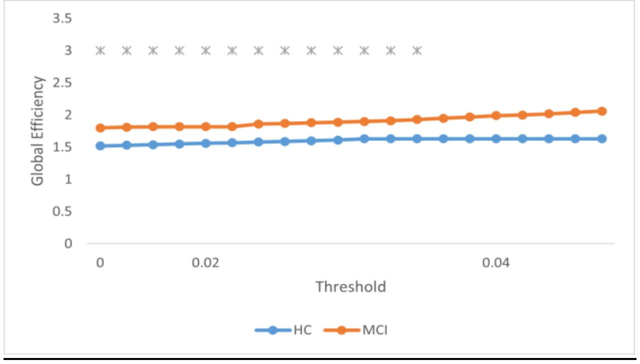

e)

Supplementary Figure 3. Group difference and network parameters comparison for HC vs. MCI under different threshold value, where figure a) Small-worldness b) mean clustering coefficient c) characteristic path length d) local efficiency and e) global efficiency. * Represents the significant group level difference with two sample t-test at P < 0.05.

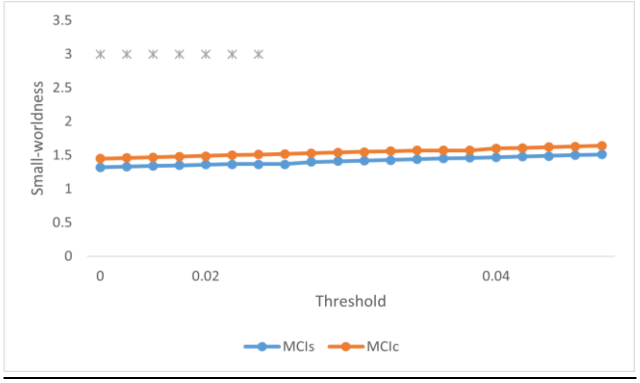

a)

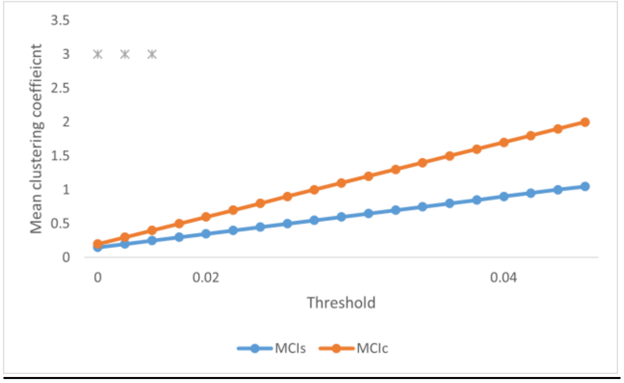

b)

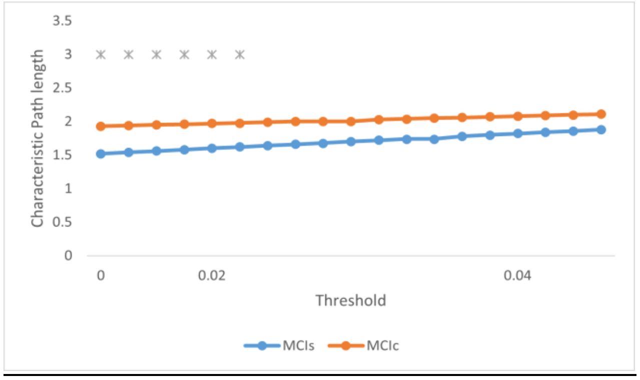

c)

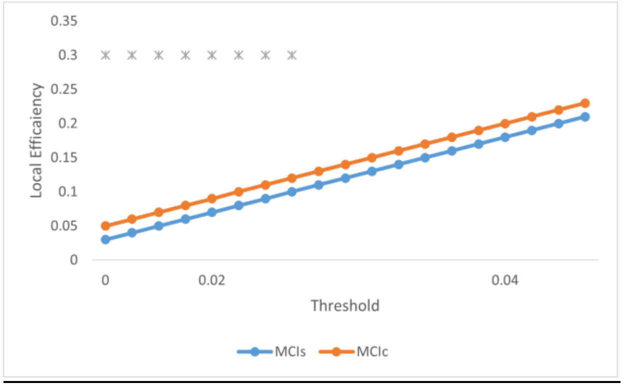

d)

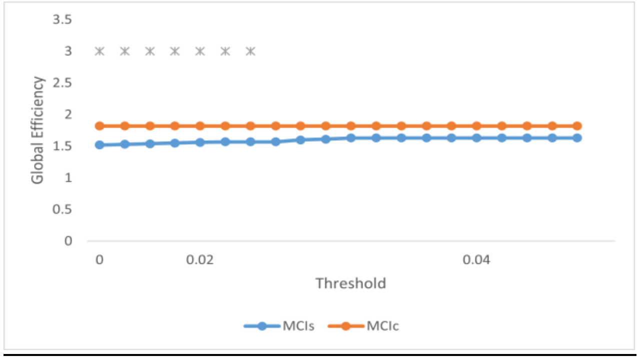

e)

Supplementary Figure 4. Group difference and network parameters comparison for MCIs vs. MCIc under different threshold value, where figure a) Small-worldness b) mean clustering coefficient c) characteristic path length d) local efficiency and e) global efficiency. * Represents the significant group level difference with two sample t-test at $P < 0.05$.

1.2 Supplementary Figures for Voxel Analysis

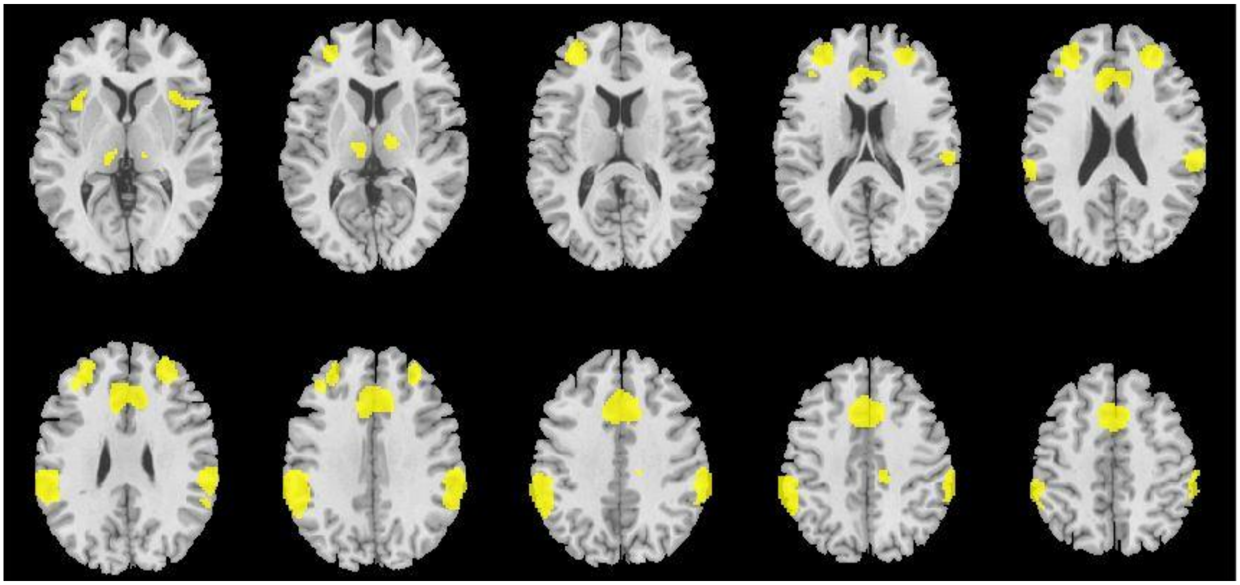

Supplementary Figure 5. The SN areas are depicted in this diagram. Shirer and his colleagues generated these templates (http://findlab.stanford.edu/functional_ROIs.html).

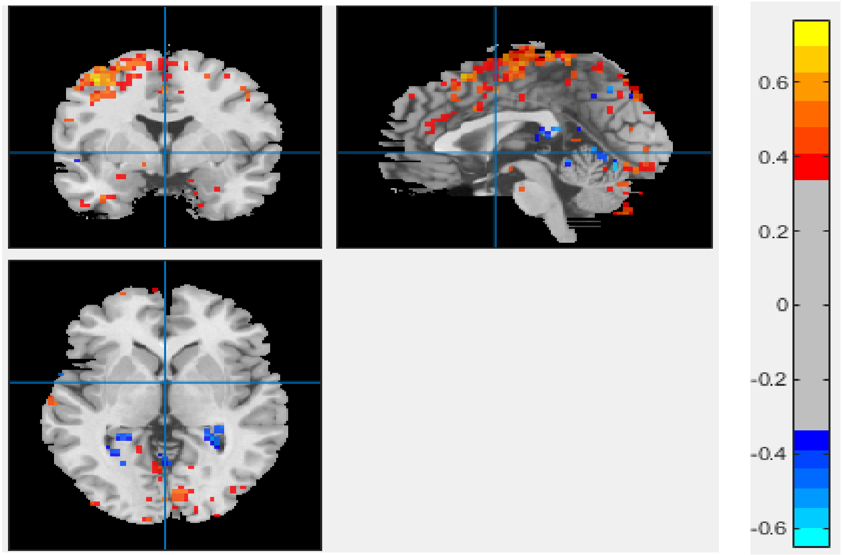

a)

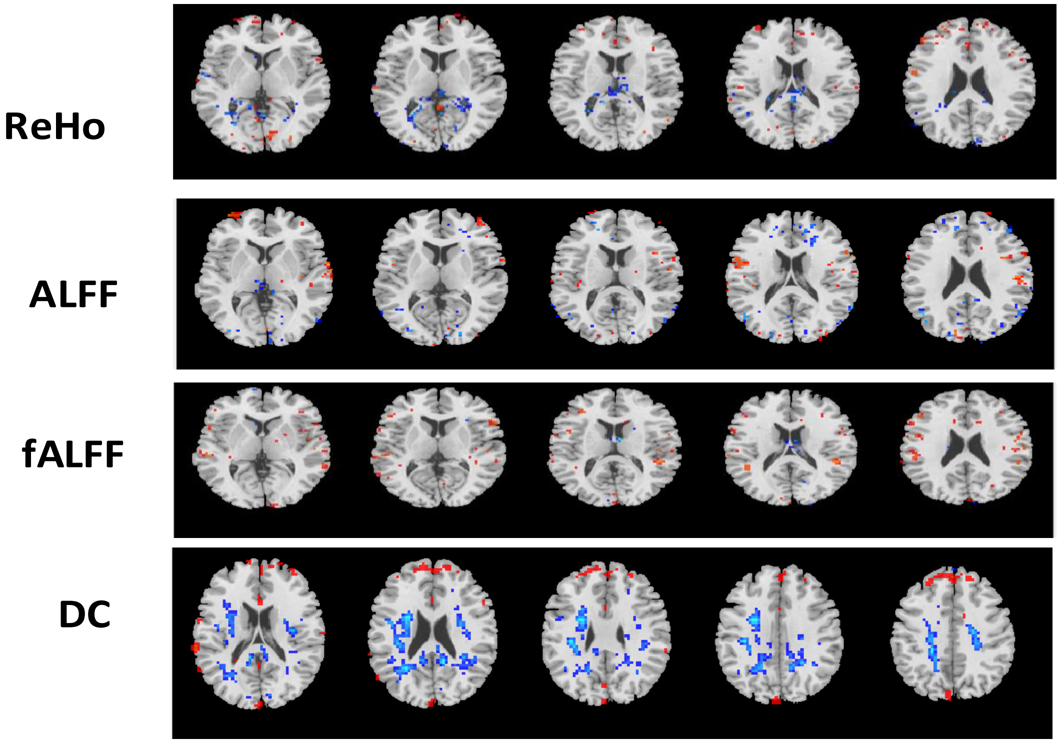

b)

Supplementary Figure 6. An univariate statistical two-sample test on ReHo voxel maps between two classification group a) AD vs MCI b) Univariate t-test difference maps between two classification group AD vs. MCI of four voxel maps. The threshold value was set to $p<0.05$. The hot and cold bar represent negative and positive changes.

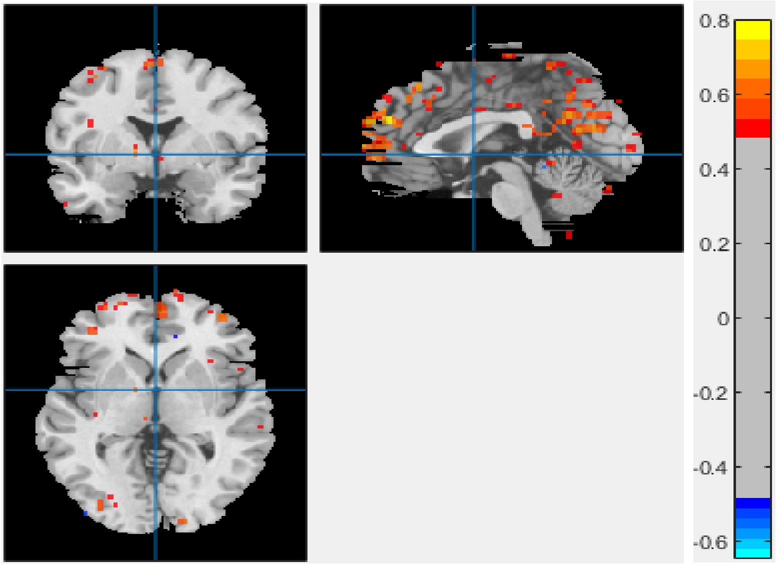

a)

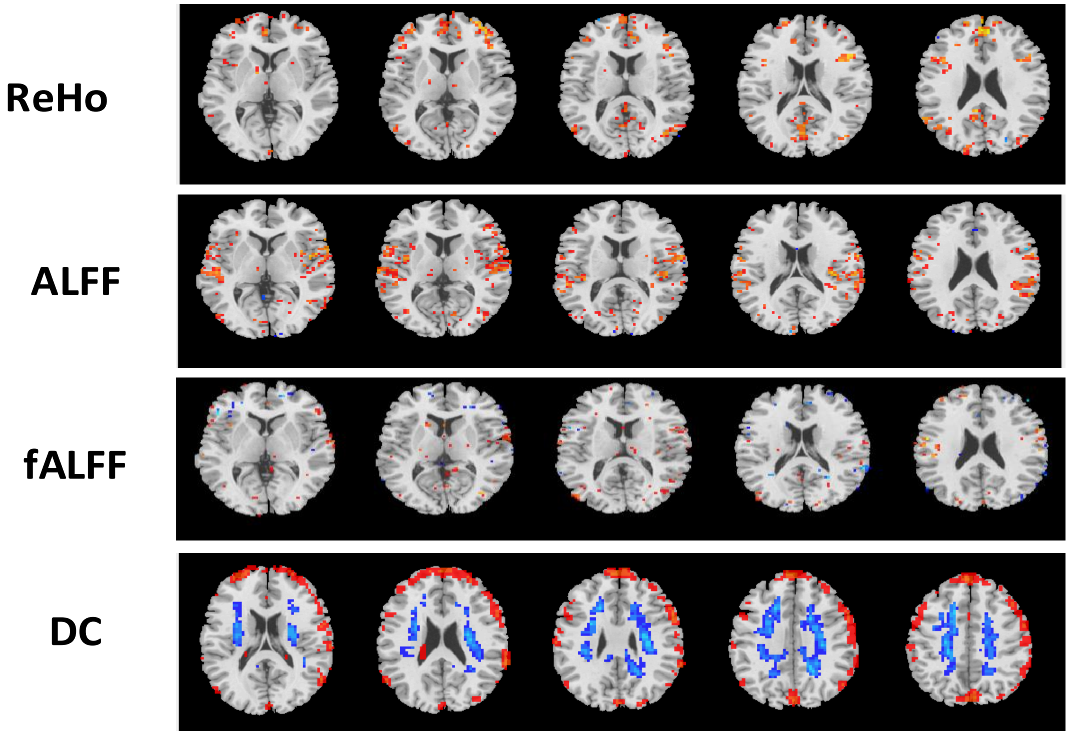

b)

Supplementary Figure 7. An univariate statistical two-sample test on ReHo voxel maps between two classification group a) HC vs MCI b) Univariate t-test difference maps between two classification group HC vs MCI of four voxel maps. The threshold value was set to $p<0.05$. The hot and cold bar represent negative and positive changes.

2 Supplementary Tables

Supplementary Table 1. The location and networks of cortical regions with highly discriminative attribute of top brain regions for AD vs HC group with their corresponding coordinate and brain regions.

ROI	AAL Regions	Networks Features	Coordinates		
			X	Y	Z
2	PreCG.R	ND	41.37	-8.21	52.09
5	ORBsup.L	BC	-16.56	47.32	-13.31
6	ORBsup.R	BC	18.49	48.1	-14.02
19	SMA.L	BC	-5.32	4.85	61.38
30	INS.R	BC	39.02	6.25	2.08
85	MTG.L	ND	-55.52	-33.8	-2.2
33	DCG.L	ND	-5.48	-14.92	41.57
35	PCG.L	BC	-4.85	-42.92	24.67
36	PCG.R	NL	7.44	-41.81	21.87
37	HIP.L	BC	-25.03	-20.74	-10.13
38	HIP.R	BC	29.23	-19.78	-10.33
41	AMYG.L	BC	-23.27	-0.67	-17.14
42	AMYG.R	BC	27.32	0.64	-17.5
45	CUN.L	ND	-5.93	-80.13	27.22
46	CUN.R	ND	13.51	-79.36	28.23
47	LING.L	BC	-14.62	-67.56	-4.63
48	LING.R	BC	16.29	-66.93	-3.87
54	IOG.R	NL	38.16	-81.99	-7.61
90	ITG.R	BC	53.69	-31.07	-22.32
32	ANG.R	BC	45.51	-59.98	38.63
67	PCUN.L	NL	-7.24	-56.07	48.01
68	PCUN.R	NL	9.98	-56.05	43.77
74	PUT.R	NL	27.78	4.91	2.46
77	THA.L	ND	-10.85	-17.56	7.98
78	THA.R	ND	13	-17.55	8.09
81	STG.L	ND	-53.16	-20.68	7.13
55	FFG.L	BC	-31.16	-40.3	-20.23
61	IPL.L	BC	-42.8	-45.82	46.74
18	ROL.R	BC	52.65	-6.25	14.63
34	DCG.R	BC	8.02	-8.83	39.79

Supplementary Table 2. The location and networks of cortical regions with highly discriminative attribute of top brain regions for AD vs MCI group with their corresponding coordinate and brain regions.

ROI	AAL Regions	Networks Features	Coordinates		
			X	Y	Z
10	ORBmid.R	BC	33.18	52.59	-10.73
21	OLF.L	BC	-8.06	15.05	-11.46
30	INS.R	BC	39.02	6.25	2.08
31	ACG.L	ND	-4.04	35.4	13.95
32	ACG.R	NL	8.46	37.01	15.84
33	DCG.R	ND	8.02	-8.83	39.79
35	PCG.L	BC	-4.85	-42.92	24.67
36	PCG.R	NL	7.44	-41.81	21.87
37	HIP.L	BC	-25.03	-20.74	-10.13
38	HIP.R	BC	29.23	-19.78	-10.33
41	AMYG.L	BC	-23.27	-0.67	-17.14
42	AMYG.R	BC	27.32	0.64	-17.5
46	CUN.L	ND	-5.93	-80.13	27.22
47	LING.L	BC	-14.62	-67.56	-4.63
48	LING.R	BC	16.29	-66.93	-3.87
63	SMG.L	NL	-55.79	-33.64	30.45
64	SMG.R	BC	57.61	-31.5	34.48
67	PCUN.L	NL	-7.24	-56.07	48.01
68	PCUN.R	NL	9.98	-56.05	43.77
78	THA.R	ND	13	-17.55	8.09
61	IPL.L	BC	-42.8	-45.82	46.74
18	ROL.R	BC	52.65	-6.25	14.63
79	HES.L	BC	-41.99	-18.88	9.98

Supplementary Table 3. The location and networks of cortical regions with highly discriminative attribute of top brain regions for HC vs MCI group with their corresponding coordinate and brain regions.

ROI	AAL Regions	Networks Features	Coordinates		
			X	Y	Z
16	ORBinf.R	ND	41.22	32.23	-11.91
19	SMA.L	BC	-5.32	4.85	61.38
28	REC.R	BC	8.35	35.64	-18.04
85	MTG.L	ND	-55.52	-33.8	-2.2
2	PreCG.R	ND	41.37	-8.21	52.09
6	ORBsup.R	BC	18.49	48.1	-14.02
30	INS.R	BC	39.02	6.25	2.08
31	ACG.L	ND	-4.04	35.4	13.95
37	HIP.L	BC	-25.03	-20.74	-10.13
38	HIP.R	BC	29.23	-19.78	-10.33
41	AMYG.L	BC	-23.27	-0.67	-17.14
42	AMYG.R	BC	27.32	0.64	-17.5
47	LING.L	BC	-14.62	-67.56	-4.63
48	LING.R	BC	16.29	-66.93	-3.87
54	IOG.R	NL	38.16	-81.99	-7.61
66	ANG.R	BC	45.51	-59.98	38.63
67	PCUN.L	NL	-7.24	-56.07	48.01
68	PCUN.R	NL	9.98	-56.05	43.77
74	PUT.R	NL	27.78	4.91	2.46
77	THA.L	ND	-10.85	-17.56	7.98
78	THA.R	ND	13	-17.55	8.09

Supplementary Table 4. The location and networks of cortical regions with highly discriminative attribute of top brain regions for MCIs vs MCIc group with their corresponding coordinate and brain regions.

ROI	AAL Regions	Networks Features	Coordinates		
			X	Y	Z
6	ORBsup.R	BC	18.49	48.1	-14.02
81	STG.L	ND	-53.16	-20.68	7.13
82	STG.R	ND	58.15	-21.78	6.8
37	HIP.L	BC	-25.03	-20.74	-10.13

38	HIP.R	BC	29.23	-19.78	-10.33
42	AMYG.R	BC	27.32	0.64	-17.5
44	CAL.R	NL	15.99	-73.15	9.4
46	CUN.R	ND	13.51	-79.36	28.23
45	CUN.L	BC	-5.93	-80.13	27.22
85	MTG.L	ND	-55.52	-33.8	-2.2
45	CUN.L	ND	-5.93	-80.13	27.22
81	STG.L	ND	-53.16	-20.68	7.13

Supplementary Table 5. Seed regions of the SN.

	Cluster	Voxels	Coordinates		
			X	Y	Z
**Anterior SN**	Frontal_Mid_L	651	-32	46	22
	Anterior Insula_L	305	-40	14	-4
	dACC	2887	0	16	46
	Frontal_Mid_R	470	28	46	26
	Anterior Insula_R	319	42	14	-2
	Cerebelum_6_L	95	-34	-56	-32
	Cerebelum_Crus1_R	139	36	-58	-32
**Posterior SN**	Frontal_Mid_L2	93	-40	36	32
	SupraMarginal_L	1205	-58	-38	36
	Precuneus_L	98	-8	-52	60
	Cingulum_Mid_R	56	12	-28	44
	Postcentral_R	133	20	-50	68
	SupraMarginal_R	1002	62	-32	42
	Thalamus_L	142	-12	-22	6
	Cerebelum_6_L2	102	-34	-42	-38
	Posterior Insula_L	114	-36	-14	-6
	Thalamus_R	63	12	-14	10
	Cerebelum_6_R	13	36	-42	-40
	Posterior Insula_R	134	40	-6	-8

Supplementary Table 6: Information for regions and peak MNI co-ordinate of Voxel-base analysis on rs-fMRI for AD vs. HC group analysis at $p<0.05$.

Cluster (T-Value)	Region	MNI Coordinate			voxels	Peak Intensity
		X	Y	Z		
**ReHo**						
**Cluster 1**	**Frontal_Mid_L**	-24	39	18	1912	9.32
**Cluster 2**	**Temporal_Mid_L**	-54	-33	6	215	5.033

Cluster 3	Temporal_Mid_R	57	-48	6	110	7.23
Cluster 4	Calcarine_L	3	-93	9	90	4.12
Cluster 5	Thalamus_L	-18	-24	3	71	8.79
Cluster 6	Cerebelum_Crusl_L	-36	-78	-27	57	3.21
Cluster 7	Thalamus_R	15	-8	12	55	5.23
Cluster 8	Temporal_Sup_R	60	-21	3	43	4.71
Cluster 9	Occipital_Mid_R	45	84	9	30	3.45
Cluster 10	Thalamus_R	18	-27	3	27	5.44
Cluster 11	Hippocampus_R	12	-33	9	22	2.3
ALFF						
Cluster 1	Hippocampus_R	27	-6	-27	408	5.56
Cluster 2	Fusiform_R	39	-39	-21	250	5.1
Cluster 3	Temporal_Inf_R	57	-3	-30	70	2.95
Cluster 4	Occipital_Sup_L	-21	-75	39	47	4.56
Cluster 5	Temporal_Mid_R	66	-18	-21	33	4.75
Cluster 6	Amygdala_L	-24	3	-18	26	4.71
Cluster 7	Cerebelum_4_5_L	-30	-33	-27	22	3.45
Cluster 8	Cerebelum_6_L	-27	-51	-18	20	2.41
FALFF						
Cluster 1	Thalamus_R	18	-12	6	57	4.25
Cluster 2	Temporal_Inf_r	51	-69	-12	33	4.03
Cluster 3	Calcarine_L	-3	-93	-9	30	3.58
Cluster 4	Precuneus_L	-15	-48	66	24	5.2
Cluster 5	Insula_L	-42	3	-9	16	5.57
DC						
Cluster 1	Frontal_Mid_L	-33	57	9	73	4.75
Cluster 2	Precuneus_R	12	-60	66	67	4.24
Cluster 3	Temporal_Mid_R	54	-42	9	55	5.13
Cluster 4	Parietal_Inf_L	-48	-48	39	50	3.23
Cluster 5	Hippocampus_L	-27	-27	-12	51	5.13
Cluster 6	cerebelum_6_R	18	-57	-27	35	4.31
Cluster 7	Occipital_Mid_R	39	-87	3	32	4.81
Cluster 9	Temporal_Mid_L	-48	-57	-3	25	4.73

Supplementary Table 7: Information for regions and peak MNI co-ordinate of Voxel-base analysis on rs-fMRI for AD vs. MCI group analysis at p<0.05.

Cluster (T-Value)	Region	MNI Coordinate			voxels	Peak Intensity
		X	Y	Z		
ReHo						
Cluster 1	Hippocampus_L	-24	-27	-6	731	5.82
Cluster 2	Precuneus_R	9	-24	42	720	4.73
Cluster 3	Hippocampus_R	36	-15	-18	670	4.57

**Cluster 4**	**Frontal_Mid_R**	27	15	48	512	4.76
**Cluster 5**	**Thalamus_L**	-15	-27	9	467	4.07
**Cluster 6**	**Cuneus_L**	3	-87	24	428	5.12
**Cluster 7**	**Precuneus_R**	9	-42	42	310	4.33
**Cluster 8**	**Pallidum_L**	-21	-3	3	180	4.31
**Cluster 9**	**Calcarine_L**	5	-60	3	73	4.01
**Cluster 10**	**Cerebelum_Crusal_L**	-39	-54	-27	67	3.43
**Cluster 11**	**Temporal_Mid_R**	57	-69	18	41	4.55
**ALFF**						
**Cluster 1**	**ParaHippocampal_L**	-21	-24	-24	875	4.58
**Cluster 2**	**Frontal_Sup_R**	15	27	36	235	5.21
**Cluster 3**	**Pallidum_L**	-21	0	-3	85	4.57
**Cluster 4**	**Lingual_R**	12	-66	0	74	4.34
**Cluster 5**	**Occipital_Mid_R**	30	-87	24	58	4.3
**Cluster 6**	**Putamen_R**	21	21	0	57	4.11
**Cluster 7**	**Thamus_R**	9	-18	0	40	4.47
**Cluster 8**	**Insula_R**	45	6	12	23	4.53
**Cluster 9**	**Calcarine_R**	6	-72	6	15	4.01
**FALFF**						
**Cluster 1**	**Precuneus_R**	6	-48	42	105	4.54
**Cluster 2**	**Occipital_Sup_L**	-12	-99	21	42	4.33
**Cluster 3**	**Thalamus_L**	-12	-21	9	35	5.38
**Cluster 4**	**Insula_L**	-39	-15	6	33	4.15
**Cluster 5**	**lingual_L**	0	-27	-24	30	4
**Cluster 6**	**Caudate_L**	-9	12	0	11	3.75
**DC**						
**Cluster 1**	**Cerebelum_8_R**	24	-54	-57	115	5.35
**Cluster 2**	**Hippocampus_R**	42	-18	-18	95	4.87
**Cluster 3**	**Cerebelum_9_L**	-12	-51	-57	83	4.77
**Cluster 4**	**ParaHippocampal_R**	18	0	-24	71	4.41
**Cluster 5**	**Fusiform_L**	-24	-3	-45	58	3.95
**Cluster 6**	**ParaHippocampal_L**	-24	0	-30	47	4.23
**Cluster 7**	**Temporal_Inf_R**	54	-48	-21	44	4.03
**Cluster 9**	**Temporal_Mid_L**	-51	-12	-21	35	3.54

Supplementary Table 8: Information for regions and peak MNI co-ordinate of Voxel-base analysis on rs-fMRI for HC vs. MCI group analysis at p<0.05.

Cluster (T-Value)	Region	MNI Coordinate			voxels	Peak Intensity
		X	Y	Z		
			**ReHo**			

**Cluster 1**	**Lingual_L**	9	-66	-9	813	4.79
**Cluster 2**	**ParaHippocampal_R**	21	9	-24	347	7.45
**Cluster 3**	**Temporal_Mid_R**	60	-15	-18	275	4.37
**Cluster 4**	**Temporal_Sup_R**	54	-12	-6	195	4.87
**Cluster 5**	**Cerebelum_Crusl_L**	-54	-54	-42	185	5.13
**Cluster 6**	**Vermis_4_5**	3	-54	-9	175	4.33
**Cluster 7**	**Cerebelum_8_L**	-27	-36	-51	150	4.31
**Cluster 8**	**Fusiform_R**	33	-75	-15	89	4.15
**Cluster 9**	**Calcarine_R**	6	-72	-9	77	3.75
**Cluster 10**	**Precuneus_L**	-9	-57	15	34	4.21
**Cluster 11**	**Amygdala_R**	24	6	-18	27	4.07
**ALFF**						
**Cluster 1**	**Frontal_Inf_Oper_R**	60	12	6	259	4.12
**Cluster 2**	**Precentral_R**	42	-9	39	230	4.55
**Cluster 3**	**Precuneus_R**	12	-54	18	95	4.73
**Cluster 4**	**Occipital_Mid_R**	42	-78	21	75	4.25
**Cluster 5**	**Insula_L**	-42	15	6	60	4.34
**Cluster 6**	**Thalamus_L**	-18	-18	9	28	3.48
**Cluster 7**	**Putamen_R**	21	6	9	23	4.12
**Cluster 8**	**Fusiform_R**	36	-30	-21	25	3.01
**Cluster 9**	**Frontal_Mid_R**	42	42	30	32	4.05
**FALFF**						
**Cluster 1**	**Lingual_L**	-30	-90	-15	78	4.31
**Cluster 2**	**Frontal_Inf_Orb_R**	48	-27	-18	66	4.54
**Cluster 3**	**Cerebelum_Crus2_R**	24	-87	-33	45	4.14
**Cluster 4**	**ParaHippocampal_L**	-21	-36	-9	43	4.35
**Cluster 5**	**Vermis_10**	0	-45	-33	31	3.75
**Cluster 6**	**Cuneus_R**	9	-87	36	27	4.7
**Cluster 7**	**Olfactory_L**	-3	21	-12	25	3.23
**DC**						
**Cluster 1**	**Hippocampus_R**	39	-30	-9	123	4.53
**Cluster 2**	**Calcarine_L**	-18	-99	-6	86	4.03
**Cluster 3**	**Frontal-Mid_Orb_L**	-33	51	-9	81	4.24
**Cluster 4**	**Fusiform_R**	33	-45	-9	53	3.57
**Cluster 5**	**Thalamus_L**	-15	-24	0	34	4.05
**Cluster 6**	**Occipital_Mid_L**	-15	-93	6	23	3.71
**Cluster 7**	**Precentral_L**	-33	-6	42	15	4.11
**Cluster 9**	**Preceneus_R**	6	-66	51	12	4.53

Supplementary Table 9: Information for regions and peak MNI co-ordinate of Voxel-base analysis on rs-fMRI for MCIs vs. MCIs group analysis at $p<0.05$.

Cluster (T-Value)	Region	MNI Coordinate			voxels	Peak Intensity
		X	Y	Z		
**ReHo**						
**Cluster 1**	**Cerebelum_6_L**	-33	-66	-21	230	4.25
**Cluster 2**	**Thalamus_L**	3	-27	15	237	4.58
**Cluster 3**	**Temporal_Inf_R**	57	-45	-24	178	5.13
**Cluster 4**	**Precuneus_R**	9	-57	-48	133	4.73
**Cluster 5**	**ParaHippocampal_R**	-21	-30	-18	93	4.55
**Cluster 6**	**Hippocampus_R**	36	-30	-12	87	4.57
**Cluster 7**	**Cerebelum-8_R**	30	-42	-48	85	4.73
**Cluster 8**	**ParaHippocampal_L**	-15	6	-27	58	4.71
**Cluster 9**	**Insula_R**	48	0	0	47	3.45
**Cluster 10**	**Temporal_Sup_L**	-51	-6	-12	24	4.03
**Cluster 11**	**Precentral_L**	-48	9	30	23	3.45
**ALFF**						
**Cluster 1**	**Lingual-R**	24	-93	-18	3790	6.45
**Cluster 2**	**Pallidum_R**	15	6	-3	750	5.73
**Cluster 3**	**Cerebelum_4_5_R**	30	-33	-33	190	4.14
**Cluster 4**	**Precentral_R**	36	-21	72	54	4.17
**Cluster 5**	**Hippocampus_L**	-24	-39	6	43	4.88
**Cluster 6**	**Occipital_Mid_L**	-27	-90	24	32	3.45
**Cluster 7**	**Fusifrom_R**	27	12	-45	30	4.56
**Cluster 8**	**Pecuneus_L**	51	-57	53	27	4.24
**Cluster 9**	**Temporal_pole_Mid_R**	54	6	-18	23	4.75
**FALFF**						
**Cluster 1**	**Hippocampus_R**	27	-30	3	87	4.37
**Cluster 2**	**Vermis_6**	6	-69	-18	59	4.04
**Cluster 3**	**Frontal_Mid_Orb_L**	-45	-48	-9	55	4.41
**Cluster 4**	**ParaHippocampal_R**	33	-33	-15	37	4.78
**Cluster 5**	**Temporal_Mid_L**	-57	3	-21	19	4.39
**Cluster 6**	**Temporal_Mid_L**	-42	6	-30	17	3.37
**DC**						
**Cluster 1**	**Fusifrom_L**	-30	-3	-45	73	4.57
**Cluster 2**	**Temp_Sup_R**	63	-6	-9	57	4.87
**Cluster 3**	**Lingual_R**	21	-60	-3	43	3.35
**Cluster 4**	**Temporal_Sup_R**	51	-24	0	18	4.58
**Cluster 5**	**Precuneus_R**	15	-39	3	13	4.07
**Cluster 6**	**Calcarine_L**	-15	-75	-6	10	3.81
**Cluster 7**	**Cuneus_L**	-9	-81	24	7	3.43
**Cluster 9**	**pariental_Inf_L**	-54	-48	45	5	4.33
